# Interaction between dry and hot extremes at a global scale using a cascade modeling framework

**DOI:** 10.1038/s41467-022-35748-7

**Published:** 2023-01-17

**Authors:** Sourav Mukherjee, Ashok Kumar Mishra, Jakob Zscheischler, Dara Entekhabi

**Affiliations:** 1 Glenn Department of Civil Engineering, Clemson University, Clemson, SC USA; 2 Department of Computational Hydrosystems, Helmholtz Centre for Environmental Research - UFZ, Leipzig, Germany; 3 Parsons Laboratory, Department of Civil and Environmental Engineering, Massachusetts Institute of Technology, Cambridge, MA 02139 USA

**Keywords:** Natural hazards, Hydrology

## Abstract

Climate change amplifies dry and hot extremes, yet the mechanism, extent, scope, and temporal scale of causal linkages between dry and hot extremes remain underexplored. Here using the concept of system dynamics, we investigate cross-scale interactions within dry-to-hot and hot-to-dry extreme event networks and quantify the magnitude, temporal-scale, and physical drivers of cascading effects (CEs) of drying-on-heating and vice-versa, across the globe. We find that locations exhibiting exceptionally strong CE (hotspots) for dry-to-hot and hot-to-dry extremes generally coincide. However, the CEs differ strongly in their timescale of interaction, hydroclimatic drivers, and sensitivity to changes in the soil-plant-atmosphere continuum and background aridity. The CE of drying-on-heating in the hotspot locations reaches its peak immediately driven by the compounding influence of vapor pressure deficit, potential evapotranspiration, and precipitation. In contrast, the CE of heating-on-drying peaks gradually dominated by concurrent changes in potential evapotranspiration, precipitation, and net-radiation with the effect of vapor pressure deficit being strongly controlled by ecosystem isohydricity and background aridity. Our results help improve our understanding of the causal linkages and the predictability of compound extremes and related impacts.

## Abstract (web summary)

This study quantifies the scope, time scale, and physical mechanisms underlying the cascade effects of drying on heating and vice versa across the various ecosystems of the world.

## Introduction

Compound dry and hot events have received much attention due to their increasing impacts on agriculture, ecosystem, health, and energy^1–6^. For instance, the 2012 summer dry and hot event in the central U.S. caused enormous economic losses of about $30 billion^7^. Among the most hazardous compound events were the dry and heat extremes that affected Europe and Russia in the summers of 2003 and 2010^8,9^ which led to massive socio-economic impacts, including around 40,000 deaths^10^, 25% loss of annual crop yield^11^, and extensive forest fires^1,12,13^. To reduce the associated potential impacts, it is essential to understand the interaction between dry and hot extreme events to aid accurate prediction and early warning systems.

Compound extreme events result from complex interactions between various physical processes across multiple spatial and temporal scales^5,6,14,15^. These interactions are characterized by the combination of various drivers, influenced by large-scale climatic processes and/or local weather systems, the impacts of which are thereby amplified^16,17^. In a multi-hazard scenario, these events can pose cascading interactions^18^. More specifically, these events are correlated through common drivers (or confounders), by virtue of which, they pose a cascading effect (CE) on one another. In practice, it is often challenging to distinguish and quantify the CEs because of model assumptions, which limit the inclusion of the complete system dynamics^16^. The relationship between hot and dry extremes is particularly intricate because of land-atmosphere feedbacks that operate at different temporal scales^19^. A precipitation deficit translates into soil-moisture depletion and positive temperature anomalies across a wide range of spatial and temporal scales. The physical processes that cause dry and hot extreme events are interrelated^6^, and, therefore, have a cascading influence on one another. Although some retrospective^20^ and empirical approaches^6,21,22^ have been applied to understand how these events propagate as cascades across the ecosystem, the extent, scope, and temporal scale of their causal interactions are not well understood. Furthermore, studies in the past did not account for the effect of other dependent variables while measuring the associations between inter-connected events, which may lead to spurious relationships and endogeneity^23^.

We assess the casual interaction between dry and hot extreme events across the globe using a probabilistic framework motivated by a system dynamics approach^24–28^. We design two independent cross-scale (temporal) interaction networks^24,27^ that represent the event-to-event (dry-to-hot and hot-to-dry event) cascades. The dry extreme events are calculated based on daily root-zone-soil-moisture (*RZSM*), whereas the hot extreme events are calculated based on daily maximum 2 m air temperature (*T*_max_) anomalies for each location across the globe. Three sets of thresholds, 1%, 5%, and 10% of daily climatological *RZSM*, and 99%, 95%, and 90% of daily climatological *T*_max_ were used to identify the dry and hot extreme events, respectively.

**Fig. 1:**
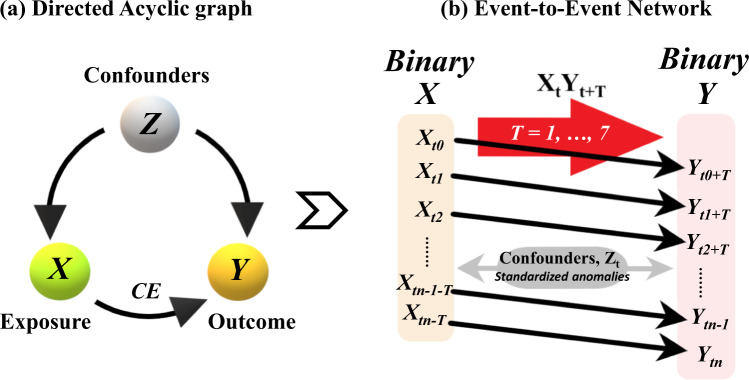
Cascade model framework. **a** directed acyclic graph representing the dynamical linkages between exposure (*X*), outcome (*Y*), and confounders (*Z*), and **b** event-to-event cascade network denoted by the cross-scale interaction, *X*_*t*_*Y*_*t+T*_ (for daily time steps, *t* = *t*_0_*, t*_1_*, …, t*_*n*_; and lag intervals, *T* = 1, ...., 7 days) for given standardized anomalies of confounders, *Z*_*t*_.

The dry and hot extreme events are subsequently considered temporal nodes of the network. These nodes are cross-linked at intervals of time lags ranging from 1-day to a week to investigate the dynamic causal effect of drying on heating and vice versa. The CEs are then quantified for each temporal network as (marginal) causal measures of association within each cascade conditioned on a set of confounders (hydroclimatic anomalies). This methodology is implemented by applying a standardized logistic regression approach^29^ centered on the concept of counterfactual probabilities (see Methods), one of the cornerstones in the modern theory of causal inference^25,26,29,30^. The main advantage of this methodology lies in its ability to robustly estimate the marginal measures of causation by isolating the main effect of one event on the other while accounting for all other dependent variables as confounders^29^. Figure 1 illustrates the directed acyclic graphs (DAGs) representing the dynamic system, and the adopted cross-scale temporal interaction network for the event-to-event cascade.

Here we investigate the cross-scale interactions within dry-to-hot and hot-to-dry extreme event networks and quantify the magnitude, temporal-scale, and physical drivers of CEs of drying-on-heating and vice versa, across the globe. More specifically, using daily RZSM data derived from the GLEAM dataset, and daily meteorological variables, maximum 2 m air temperature (*T*_max_), total precipitation (*Pr*), vapor pressure deficit (VPD), potential evapotranspiration (PET), and net surface radiation (*Rn*) derived from the European Centre for Medium‐Range Weather Forecasts Reanalysis 5 (*ERA5*), and vegetation optical depth (VOD) data from the global land parameter data record (LPDR) version 3^31^, we aim to answer the following questions: what are the hotspots of cascading dry and hot extreme events across the globe? How do the key hydroclimatic variables influence the cascading association between dry and hot extreme events? And what is the role of the soil–plant–atmosphere continuum and background aridity in influencing the cascading causal interactions? The results from the study highlight the global hotspots and characterize the scale of cascading interactions between dry and hot extreme events, which is likely to aid in the quantification of the risk of crop-yield losses, wildfires, and water scarcity across the globe. The underlying mechanisms are also investigated by exploring the potential influence of hydroclimatic anomalies and the role of surface energy partitioning on the causal linkages between terrestrial drying and heating.

## Results

### Hotspots of cascading dry and hot extremes

The CE of dry-to-hot and hot-to-dry extreme event occurrence is quantified based on the magnitude of attributable fraction (AF)^32^. A data-driven cascade model framework is implemented using daily dry-to-hot and hot-to-dry extreme event networks for the 1980–2018 period considering multiple time-lags, *T* = 1–7, and multiple confounders. Dry and hot extreme events are identified based on three different daily climatological thresholds of *RZSM* (1, 5, and 10%) and *T*_max_, (99, 95, and 90%), respectively. Based on three different combinations of thresholds, three different dry-to-hot extreme event cascades are derived, referred to as *D1pH99p*, *D5pH95p*, and *D10pH90p*, where *p* stands for percentile. Similarly, three different hot-to-dry extreme event cascades are derived, which are referred to as *H99pD1p*, *H95pD5p*, and *H90pD10p*.

For each of the event cascades, AF is calculated for all grid cells based on the dry-to-hot and hot-to-dry event networks by applying a regression standardization technique^30^ for each of the selected time-lags, *T* (hereafter referred to as AF_*T*_), separately. The regression standardization is helpful for obtaining robust marginal measures of association between the exposure and outcome variable by accounting for all other variables as confounders. Thus, AF_*T*_ (%) for the dry-to-hot (as *D1pH99p, D5pH95p*, or *D10pH90p*) extreme event cascade represents the standardized risk of having a hot extreme event caused by a dry extreme event that occurred *T*-days before. Similarly, AF_*T*_ (%) for the hot-to-dry (*H99pD1p*, *H95pD5p*, or *H90pD10p*) extreme event cascade represents the standardized risk of having a dry extreme event caused by a hot extreme event that occurred *T* days before. In other words, AF_*T*_ determines the strength of the CE of drying on heating and vice versa. The cascade model framework, implementation, and association with selected confounders are presented in Fig. 1. A detailed discussion on how the regression standardization is implemented to account for the dependence of the exposure-to-outcome (*X*_*t*_*-Y*_*t+T*_) relationship on the confounding variables (*Z*_*t*_) is provided in the “Methods”.

**Fig. 2:**
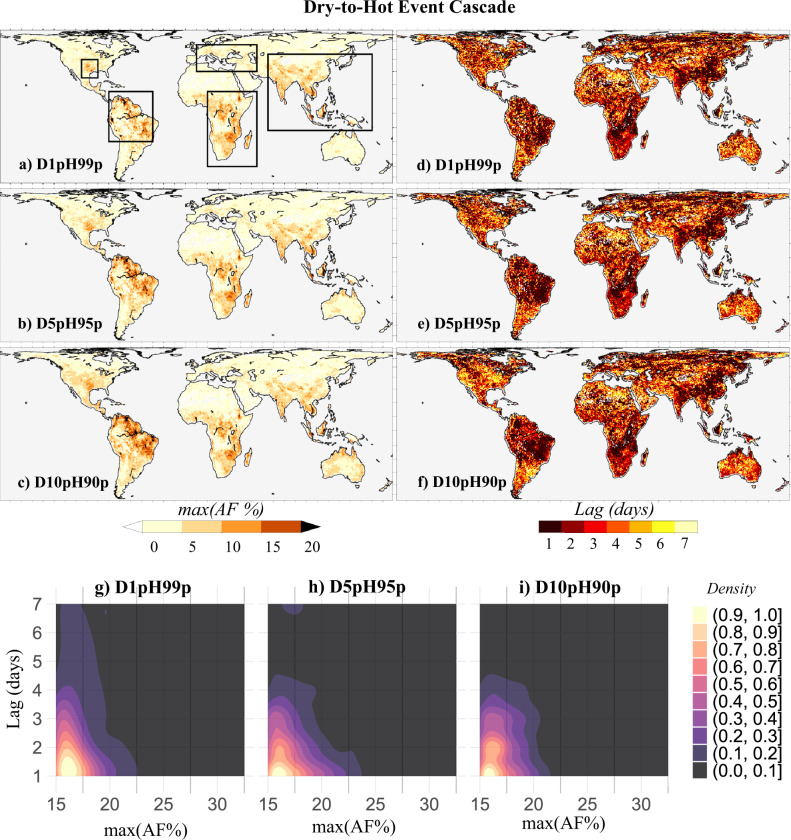
Hotspots of dry-to-hot extreme event cascade. Spatial map of **a**–**c** maximum attributable fraction (AF) magnitudes that are statistically significant at 95% confidence level for the drought-heatwave cascade derived using (**a**) 1 percentile of daily root-zone-soil-moisture (RZSM), and 99th percentile of daily maximum temperature (*T*_max_) as threshold (D1pH99p), **b** 5th percentile of RZSM, and 95th percentile of *T*_max_ as threshold (D5pH95p), and **c** 10th percentile of RZSM, and 90th percentile of *T*_max_ as threshold (D10pH90p), calculated for the period, 1980–2018, and **d**–**f** corresponding lags for which the maximum AF magnitudes (shown in **a**–**c**) are observed (**g**, **h**) bivariate kernel density estimates (probability density shown by shading) calculated for the grid-point maxima of AF magnitudes and corresponding lag-times considering all grids identified as hotspots (AF_*T*_ ≥ 15%).

**Fig. 3:**
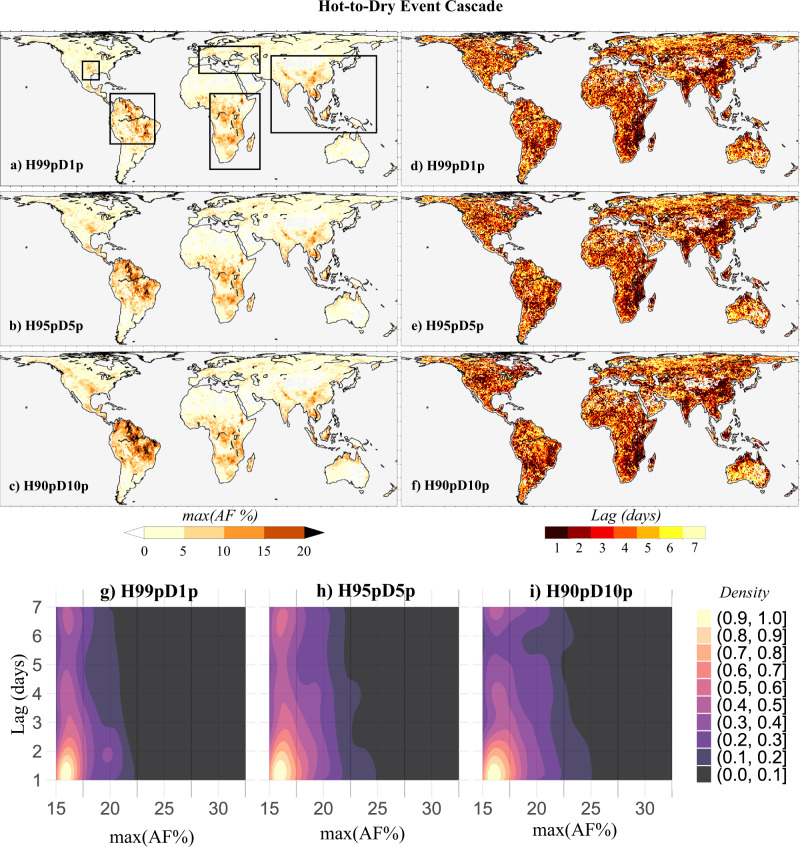
Hotspots of hot-to-dry extreme event cascade. Spatial map of **a**–**c** maximum attributable fraction (AF) magnitudes that are statistically significant at 95% confidence level for the heatwave-drought cascade derived using **a** 1 percentile of RZSM, and 99th percentile of *T*_max_ as threshold (H99pD1p), **b** 5th percentile of RZSM, and 95th percentile of Tmax as threshold (H95pD5p), and **c** 10th percentile of RZSM, and 90th percentile of Tmax as threshold (H90pD10p), calculated for the period, 1980–2018, and **d**–**f** corresponding lags for which the maximum AF magnitudes (shown in **a**–**c**) are observed, **g**, **h** bivariate kernel density estimates (probability density shown by shading) calculated for the grid-point maxima of AF magnitudes and corresponding lag-times considering all grids identified as hotspots (AF_*T*_ ≥ 15%).

To illustrate the hotspots of CE of both drying on heating and heating on drying, we calculated the maximum AF_*T*_ ( = max{AF_0_, AF_1_, AF_2_, AF_3_, AF_4_, AF_5_, AF_6_, AF_7_}) across each (0.5° × 0.5°) pixel of the globe for all the selected dry-to-hot and hot-to-dry extreme event cascades. Note that the maximum value of the *AF* is calculated from the AF_*T*_ values that are statistically significant. The statistical significance of the AF_*T*_ values is determined based on their 2.5–97.5% confidence interval (see Methods). The estimates, lower and upper bounds (at 95% confidence level) of AF_*T*_ for each of the lag timings (*T* = 1–7 days) corresponding to the selected cascades are presented in Supplementary Figs. 1–6. The key statistics of the global distribution of the maximum (and statistically significant) AF_*T*_ are illustrated by boxplots in Supplementary Fig. 7 for the selected cascades. Based on these statistics, the maximum AF_*T*_ magnitudes are further classified into four different categories, moderate (0 ≤ AF_*T*_ < 5%), severe (5 ≤ AF_*T*_ < 10%), extreme (10 ≤ AF_*T*_ < 15%), and exceptional (AF_*T*_ ≥ 15%). Thus, a maximum AF_*T*_ ≥ 15%, falling in the exceptional category, for any given dry-to-hot cascade indicates that more than 15% of the extreme hot events are caused by extreme dry events that occurred at a lag of *T* days. We define a given pixel as a hotspot if the corresponding value of maximum AF_*T*_ falls within the extreme (10 ≤ AF_*T*_ < 15%) to exceptional (AF_*T*_ ≥ 15%) category range. Figures 2a–c and 3a–c demonstrate the spatial distribution of the maximum AF_*T*_ values and the hotspots of CE for the dry-to-hot and hot-to-dry extreme event cascades over the globe, respectively. The corresponding number of lags for which the AF_*T*_ is found to be maximum is noted for each pixel as presented in Figs. 2d–f and 3d–f.

Figures 2a–c and 3a–c suggests that extreme to exceptionally (AF_*T*_ > 10%) strong CE of both dry-to-hot and hot-to-dry extreme event are prominent over the lower Mississippi river basin located in the southern US, major parts of the Amazon River basin located in the northern South American continent, central and southern Africa, central and southern Europe, and central, east, and south Asia. These hotspot regions (delineated by bold lines in Fig. 2a) are consistent across the three hot-to-dry (*D1pH99p, D5pH95p, and D10pH90p*) and hot-to-dry (*H99pD1p, H95pD5p*, or *H90pD10p*) extreme event cascades. However, the CE for the dry-to-hot and hot-to-dry extreme events cascades vary strongly in the timescale of their causal linkages, especially in the hotspot locations (Figs. 2d–f and 3d–f). This asymmetry in the timescale of interaction is notable from the bivariate probability density estimates shown in Figs. 2g–i and 3g–i. While there is a higher probability that CE for the dry-to-hot extreme event cascades in the hotspot regions is highest for a time-lag of 1 day, that of the hot-to-dry extreme cascades is highest for the time-lag between 2 and 7 days. Furthermore, the corresponding lags for which the maximum AF magnitudes (shown in a-c) are observed are found to be more spatially heterogeneous in the case of the hot-to-dry cascade compared to the dry-to-hot cascade. This spatial heterogeneity may be linked to the underlying effect of confounders and their interactions.

Overall, the hotspot CE for both dry-to-hot and hot-to-dry cascade networks closely matches the spatial locations. Nevertheless, the differences in the temporal scale of the causal associations observed for the dry-to-hot and hot-to-dry extreme event cascades may be associated with the persistency of SM^33,34^. A higher soil moisture autocorrelation reflects that soil moisture anomaly is more persistent^35^. To investigate the association between soil moisture memory and the temporal scale of CEs, we calculated the grid-point average of the one-month lagged autocorrelation coefficient (*ρ*) of monthly RZSM anomalies (1980–2018) specific to each lag day (from 1 day to 7 days) for which the maximum CEs are obtained for the dry-to-hot and hot-to-dry events, separately (as shown in Supplementary Fig. 8). For instance, the grid-point average of ρ specific to 1-day lag is calculated by considering all grid locations that show the strongest CEs for the cascade with 1-day lag. To remove seasonality, the monthly anomalies of RZSM are obtained by subtracting the long-term monthly climatology (1980–2018) from the observed series. A higher autocorrelation is noted for higher lag (7 days) in the case of hot-to-dry events, and for lower lag (1 day) in the case of dry-to-hot events (Supplementary Fig. 8). These results indicate that, while dry soils, through surface energy partitioning, tend to affect air temperatures immediately, hot air takes a few days to cause soil desiccation due to SM memory, particularly when considering the deeper soil layers (root-zone)^36–38^.

### Influence of hydroclimatic anomalies

The influence of hydroclimatic anomalies (or confounders) on the CE of dry-to-hot and hot-to-dry extreme event cascades is investigated based on odd ratios calculated by fitting a logit model, embedded within the cascade model framework (see Methods). The odd ratios for the dry-to-hot event cascade are calculated by fitting the logistic regression using the binary sequence of extreme dry days as the independent variable (*X*) and that of extremely hot days as the dependent variable (*Y*) with *Z* as the confounding variable. On the other hand, the odd ratios for the hot-to-dry event cascade are calculated similarly but using the binary sequence of extremely hot days as the independent variable (*X*) and extremely dry days as the dependent variable (*Y*). The Odd ratio is given as exp(*β*), where *β* is the regression coefficient of the logit model, such that exp(*β*) > 1 and exp(*β*) < 1 indicate a multiplicative increase and decrease, respectively, in the daily odds of an outcome for a given exposure and per unit increase in the hydroclimatic variables or confounders (here, standardized anomalies of PET, VPD, Pr, and Rn), measured for specific time lags. For example, an odd ratio of 1.2 for a given dry-to-hot event cascade with a temporal scale of *T* days implies that for per unit increase in the confounder variable, the odds of occurrence of an extreme hot event are likely to increase by 1.2 times given a dry extreme event has occurred at a time-lag of *T* days.

**Fig. 4:**
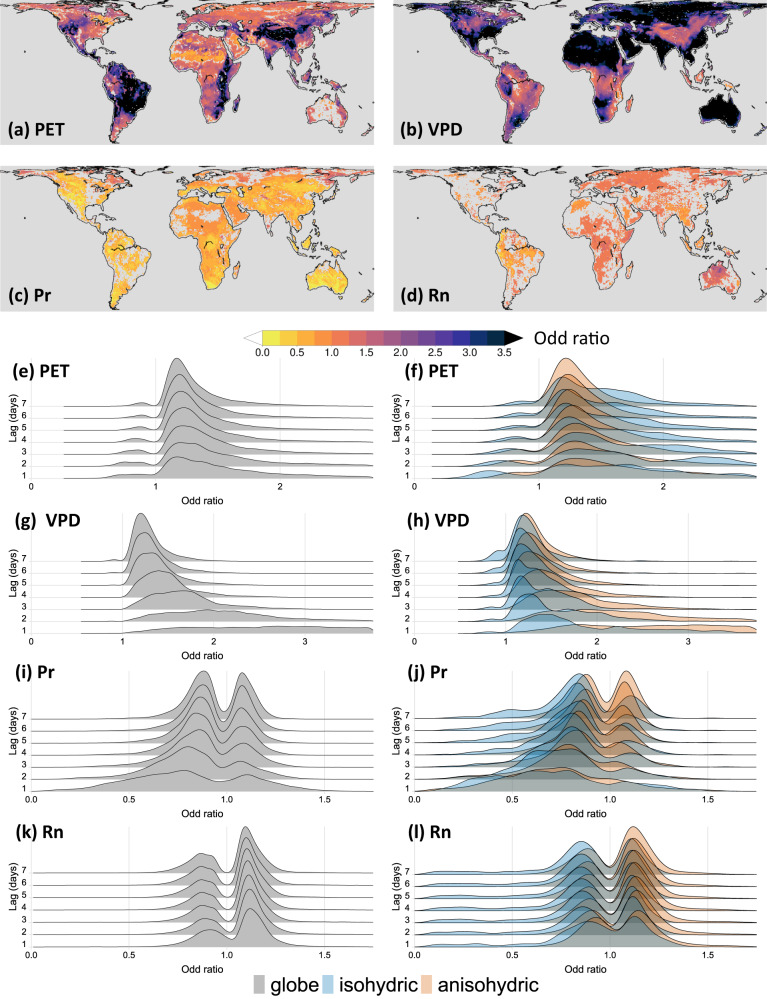
Influence of hydroclimatic anomalies for dry-to-hot extreme event cascade. **a**–**d** Spatial maps of statistically significant (at 95% confidence level) odd ratios (exp(*β*)) calculated by fitting the logistic regression model for the selected dry-to-hot (*D1pH99p*) event cascade for time-lag of 1 day associated with **a** potential evapotranspiration (PET), **b** vapor pressure deficit (VPD), **c** precipitation (Pr), and **d** net radiation (Rn), **e**, **f** probability distribution function of statistically significant odd ratios for PET in the *D1pH99p* event cascade for time-lags ranging from 1 to 7 days for the **e** global, and **f** strongly isohydric (*σ* < 0.1) and strongly anisohydric (*σ* > 0.9) region, **g**, **h** same as in (**e**, **f**) but for VPD, **i**, **j** same as in (**e**, **f**) but for *Pr*, **k**, **l** same as in (**e**, **f**) but for *Rn*. Note that these probability distribution functions are derived based on non-parametric kernel density estimates.

**Fig. 5:**
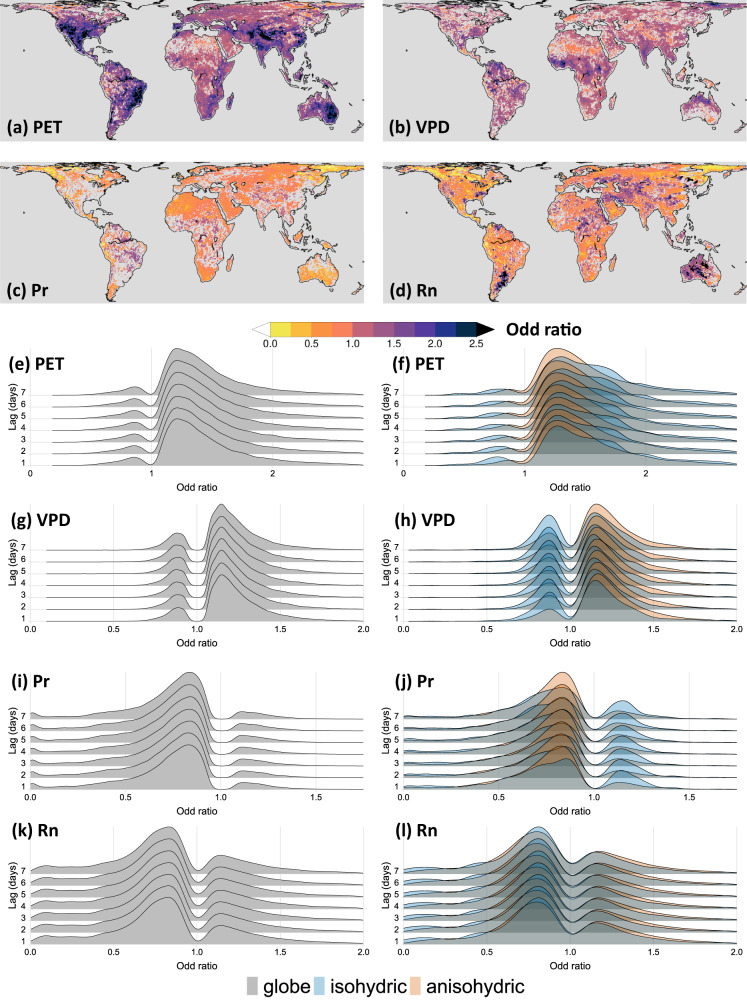
Influence of hydroclimatic anomalies for hot-to-dry extreme event cascade. **a**–**d** Spatial maps of statistically significant (at 95% confidence level) odd ratios (exp(*β*)) calculated by fitting the logistic regression model for the selected hot-to-dry (*H99pD1p*) event cascade for time-lag of 1 day associated with **a** potential evapotranspiration (PET), **b** vapor pressure deficit (VPD), **c** precipitation (Pr), and **d** net radiation (Rn), **e**, **f** probability distribution function of statistically significant odd ratios for PET in the *H99pD1p* event cascade for time-lags ranging from 1 to 7 days for the **e** global, and **f** strongly isohydric (*σ* < 0.1) and strongly anisohydric (*σ* > 0.9) region, **g**, **h** same as in (**e**, **f**) but for VPD, **i**, **j** same as in (**e**, **f**) but for *Pr*, **k**, **l** same as in (**e**, **f**) but for *Rn*. Note that these probability distribution functions are derived based on non-parametric kernel density estimates.

Figures 4 and 5, and Supplementary Figs. 9 and 20 illustrate the spatial distribution of statistically significant (at 95% confidence level) odd ratio corresponding to PET, VPD, Pr, and Rn for the dry-to-hot (*D1pH99p, D5pH95p, and D10pH90p*) and hot-to-dry (*H99pD1p, H95pD5p*, or *H90pD10p*) extreme event cascades for 1 day, and 2- to 7-day lags, respectively. The spatial distribution of the odd ratios (exp(*β*)) for the *D1pH99p* (dry-to-hot) and *H99pD1p* (hot-to-dry) event cascades for each time-lag is further summarized by deriving the non-parametric kernel density estimates considering the global grid-points as shown in Figs. 4, 5, respectively. Plants are known to respond differently to high VPD, conceptualized as isohydricity^39^, an important feature of the soil–plant–atmosphere-continuum. To investigate the effect of ecosystem-scale plant isohydricity on the odd ratios, the kernel density estimates are evaluated separately considering the grid points where the plant species exhibit strong isohydricity (*σ* < 0.1) and anisohydricity (*σ* > 0.9; see Methods and Supplementary Fig. 21).

Figures 4 and 5 and Supplementary Figs. 9 and 20 suggest in each location, the influence of one or more confounding variables shows a dominant influence of others. This also indicates the effect of interaction among the confounding variables. Such interactions vary spatially across the globe. For example, the positive influence of PET on the odds of the dry-to-hot cascade, with a 1-day lag, is substantially higher (odd ratio > 3) in the semi-arid and sub-humid regions of central and east Asia, western North America, northern and central South America, and south-eastern Africa (Fig. 4a, b). In most of these regions, VPD shows a positive but moderate (1 < odd ratio < 2), Pr show a negative but weaker (0.5 < odd ratio < 1), and Rn show a negligible influence. On the other hand, VPD show relatively more dominant influence (odd ratio > 3) in the majority of the globe, including the northern, southern, and southeastern North America, part of southeastern South America, arid and semi-arid regions of northern and southwestern Africa, middle east, southern and eastern Europe, Australia, northern and eastern Asia, including the majority of Russia, China, and India. Some of these regions show compounding influence of VPD and Pr, such as in Australia, and the southernmost parts of Africa, where the values of odd ratio indicate that a simultaneous decrease in Pr and increase in VPD anomalies by one unit can yield 3-4 times increase in the odds of dry-to-hot event cascade in these regions. The influence of PET is relatively weaker and even shows a negative impact on the odds of the occurrence of dry-to-hot cascade in some of these regions, especially in the arid regions of Australia, Southern, and northern Africa, and some parts of the middle east.

A similar interaction of the influence of confounding variables, PET, VPD, Pr, and Rn can be observed for the hot-to-dry event cascade (Fig. 5a–d, and Supplementary Figs. 9 and 20). For example, the relative dominance of PET over the influence of Pr is noteworthy in the majority of central and southern North America, eastern Australia, and east Africa. In these regions, the odd ratio for PET is found to be greater than 2, whereas the odd ratio for Pr is found to be between 0.75 and 1. Interestingly, in the humid regions of South America, central Africa, and arid and semi-arid regions of central and eastern Asia, where PET show dominant influence (odd ratio > 2), Pr shows weak positive influence (1 < odd ratio < 1.5) on the odds of hot-to-dry event cascade. This is contrasting with the strong negative influence of Pr and the weak but negative influence of PET found in the northeastern parts of Asia, northwestern parts of North America, arid regions of North Africa, and some parts of the western Amazon basin.

During extreme drought conditions, limited soil moisture availability progressively reduces land evaporation, after which any incoming radiation leads to an increase in sensible heating of the near-surface atmosphere that often develops into a heatwave^19^. This justifies the weak and negative influence of PET on the odds of dry-to-hot and hot-to-dry cascade in locations where Pr shows a more dominant negative effect^19^. The influence of Rn is relatively more spatially heterogenous with a stronger negative influence over the majority of North America and northern Russia, whereas Pr also shows a strong negative influence on the odds of hot-to-dry events (Fig. 5 and Supplementary Figs. 18–20). This is possible during a drought–heatwave–drought cascade when a reduction in rainfall increases the albedo of the exposed surface resulting in a decrease in Rn^40^.

The effect of VPD is strongest for a lag of 1 day in case of a dry-to-hot cascade, and its influence weakens significantly for an increasing number of lags from 1 to 7 days (Fig. 4e–l). Although the influence of PET and Pr is also relatively stronger for shorter time lags, their influence is still weaker compared to VPD. These results suggest that increases in VPD are more likely associated with a more spatially dominant and immediate increase in the odds of dry-to-hot extreme event cascade. Unlike PET, Pr, and Rn, which essentially describe local water and energy fluxes, VPD informs on the aridity of air and confounds ecosystem dryness stress through strong coupling with soil moisture^41,42^. As such, increasing VPD implies increasing atmospheric moisture demand, but is also a testament to the simultaneous failure of surface evaporation to meet this demand. The instantaneous response is thus fully consistent with what is expected from already hot and dry air masses interacting with desiccating soils, and an ensuing shift toward even stronger surface sensible heating.

The influence of VPD in the case of hot-to-dry events is relatively weaker in the isohydric regions. These regions mostly exhibit a decrease in the CE of heating on drying with the increase in VPD (Fig. 5). This is possible during heatwaves at instances of elevated VPD that is responsible for decoupling the sensitivity of the stomatal functions and leaf water potential (*Ψ*_L_) to changes in the soil water potential (*Ψ*_S_)^39^. This type of decoupling is more common in isohydric species due to a strong reduction in stomatal conductance during heat waves. This explains why even during substantially high atmospheric moisture deficits, the soil moisture stress in the isohydric regions may still remain low^39^. In addition, the negative effect of PET on the CE of drying on heating and heating on drying is more pronounced in the isohydric species and depends on the water use efficiency during drought. This is because relatively isohydric species strongly resist stomatal functions during drought, thereby progressively reducing the relative contribution of transpiration to evaporation^43^. It should be noted that an odd ratio = 1 is nonsignificant and thus eliminated from the estimation of probability densities, which is why the probability distributions of statistically significant odd ratios show bimodality (Figs. 4 and 5).

Overall, considerable spatial heterogeneity is noted for the influence of hydroclimatic variables on the hot-to-dry event cascades. This explains why relatively more spatial heterogeneity is observed for the lags corresponding to the strongest CE of the hot-to-dry cascade (Fig. 3d–f). These results suggest that the confounding influence of daily climate variables can have a spatially disproportionate effect on the dry-to-hot and hot-to-dry event cascade across the terrestrial surface. Such spatial heterogeneity may arise from variation in surface energy partitioning^40^, which is mainly controlled by the background aridity of a region^5,44^.

### Role of background aridity

The background aridity of a region plays a critical role in controlling the water use effciency^45^, the sensitivity of evaporation to changes in temperature and precipitation^5,46^, and causal interactions between precipitation, evaporation, and soil moisture^47^. We investigate the control of background aridity on the CE in dry-to-hot and hot-to-dry event cascades separately to understand the implication of surface energy partitioning for each of these cascades.

**Fig. 6:**
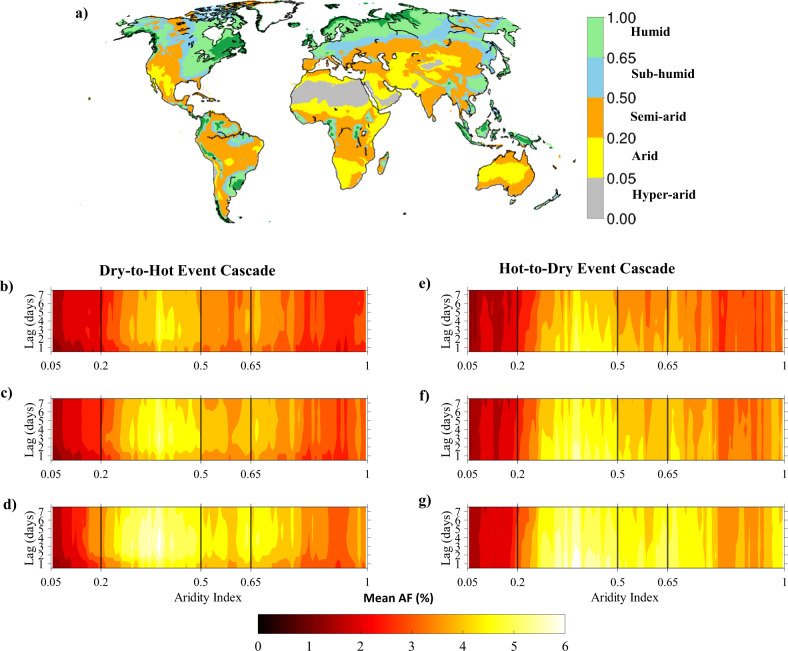
Sensitivity of cascading effect to change in background aridity. **a** Spatial map showing the classification of global evaporation regimes based on aridity index (AI) calculated as a ratio of mean annual precipitation and potential evaporation for the 1980–2018 period, **b**–**d** mean of statistically significant (at 95% confidence level) AF (%) binned as a function of AI and time-lags (1–7 days) for the three types of dry-to-hot event cascade, **b** *D1pH99p*, **c** *D5pH95p*, and **d** *D10pH90p*, and **e**–**g** same as in (**b**–**d**) but for the three types of hot-to-dry event cascade, **e** *H99pD1p*, **f** *H95pD5p*, and **g** *H90pD10p*, selected for the study.

**Fig. 7:**
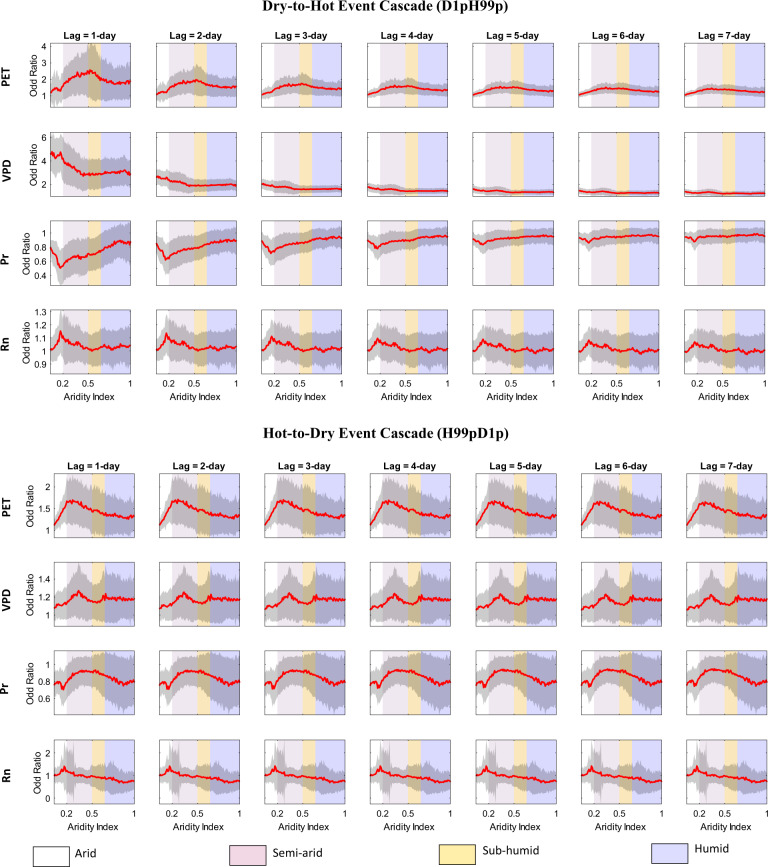
Sensitivity of hydroclimatic influence to change in background aridity. Variation of grid-point average (bold line in red), and one standard deviation (shading in black) of odd ratios for potential evapotranspiration (PET), vapor pressure deficit (VPD), precipitation (Pr), and net radiation (Rn), corresponding to the occurrence of dry-to-hot event cascade, D1pH99p (top panels), and hot-to-dry event cascade, H99pD1p (for 1- to 7-day lag) with change in Aridity index. The shading in the background indicates the different evaporation regimes.

Background aridity is quantified based on aridity index (AI)^48^, calculated as a ratio between annual climatological mean precipitation and annual climatological mean potential evaporation for the 1980-2018 period obtained from the ERA5 dataset. The global regions are then subdivided into hyper-arid, arid, semi-arid, sub-humid, and humid regimes following an AI-based classification system proposed by United Nations Environment Program^48^. The five climate regimes and the corresponding range of AI are illustrated in Fig. 6a. To investigate the role of background aridity on the CEs and the influence of confounders in the dry-to-hot and hot-to-dry extreme event cascades, we considered the AI range (0.05–1) starting from the arid to humid regimes and divided the globe into 96 sub-regimes at intervals of 0.01. The corresponding pixels within the sub-regions were extracted, and the magnitude of AF (%) and odd ratios of PET, VPD, PR, and Rn, which are statistically significant (at 95% confidence level), were averaged across those pixels for each cascade. Figure 6b–g demonstrates the magnitude of mean AF (represented by shading) varying from the arid (AI < 0.20) to humid (AI > 0.65) regimes for different time lags (represented in the *y*-axis) corresponding to the dry-to-hot (*D1pH99p*, *D5pH95p*, *and D10pH90p*) and hot-to-dry (*H99pD1p*, *H95pD5p*, or *H90pD10p*) extreme event cascades. Figure 7 shows the variation of odd ratios of PET, VPD, Pr, and Rn with AI for the lag of 1–7 days for the D1pH99p, and H99pD1p event cascades.

Dominant control of background aridity on the CE in both dry-to-hot and hot-to-dry event cascades is exhibited by the variation of the mean values of AF across the evaporation regimes for the different combinations of selected thresholds (of *RZSM* and *T*_max_) and temporal lags. For example, a relatively higher mean CE (with mean AF between 4.5 and 6%) is noted across the semi-arid (0.20 ≤ AI ≤ 0.50) regions of the globe for all events. Interestingly, stronger CE is noted for the D1pH99p and H99pD1p event cascade across the semi-arid regimes. A similar strong CE is noted across the transitional (semi-arid and sub-humid) regimes (0.20 ≤ AI ≤ 0.65) for the less severe dry-to-hot cascades (D5pH95p and D10pH90p) and hot-to-dry cascades (H95pD5p and H90pD10p) for time-lags up to 7 days. These results underscore the non-linear control of surface energy partitioning on causal interaction between drying and heating of the terrestrial surface. Relatively stronger CE of drying on heating can be noted in the semi-arid, and sub-humid regimes. Semi-arid and sub-humid regimes that transition between wet to dry conditions are suggested as the hotspots for strong soil moisture–evaporation–temperature as well as soil moisture-evaporation-precipitation coupling^19,49–51^. During drought, limited water availability reduces surface evaporation, thereby limiting latent heat fluxes, which leads to a systematic increase in sensible heating. Such feedback between soil moisture-evaporation-temperature is common in anticyclonic conditions that often provide enough time for heatwaves to develop. In transition zones, evaporation is high enough to trigger moist convection from boundary layer moisture but is dominated by the variation in soil moisture. This leads to stronger precipitation-soil moisture feedbacks, which are expected to further enhance the drying leading to the onset of drought-heatwave cascade^19,51^. Similarly, increased advection during a heatwave and mega-heatwave events can reduce land evaporation, often contributing to soil moisture drought^8,52,53^. During strong heatwaves governed by anticyclones in the midlatitudes, and sufficiently far from the center of the anticyclone where advection is weak, there can be advection of hot and dry upwind which will promote (bare-soil) evaporation and hence enable even faster soil desiccation^53^. Moreover, in transitional regimes, sometimes due to limited albedo effect from vegetation dieback and exposed soil from existing drought, the energy partitioning effect becomes a more dominant factor than net radiation in influencing drought-heatwave cascade^40^.

The impact of the confounding variables, PET, VPD, Pr, and Rn, on the CE of the dry-to-hot and hot-to-dry event, show considerable sensitivity to changes in background aridity. This is indicated by the variation in odd ratios of PET, VPD, PR, and Rn for the dry-to-hot and hot-to-dry cascade events (Fig. 7). In the case of dry-to-hot event cascades, this variation is considerably higher for shorter time lags. For the 1-day lag, when the CE of dry-to-hot events is generally greatest (Fig. 3), the odd ratio associated with PET shows a steady increase with the increase in AI from 0.2 in the semi-arid regime until it peaks in the sub-humid regime and then decreases with further increase in AI from 0.55 to 1 in the humid regimes. A simultaneous and steady increase (decrease) in the odd ratio of Pr (VPD) is observed from AI > 0.2 in the semi-arid regimes up to AI ≤ 0.75 in the humid regimes, indicating a weaker influence of Pr (VPD) on the CE of drying on heating in the sub-humid and humid regime compared to the arid region. In the case of a hot-to-dry event cascade, the variation of the odd ratio with AI is high but consistent for all time lags (Fig. 7). The variation of the odd ratio of PET, VPD, and Pr with background aridity in the case of hot-to-dry cascade is also simultaneous. However, these variations are notably different compared to the dry-to-hot cascade. CE of the hot-to-dry cascade is relatively more sensitive to changes in PET, and VPD in the semi-arid regimes. On the other hand, Pr and Rn both show a relatively higher sensitivity in the semi-arid, and humid regions.

The stronger influence of PET in the semi-arid and sub-humid regimes is likely due to stronger soil moisture-evaporation-temperature coupling, typically observed in these regions^50,54^. During soil moisture drought, precipitation can occur in moderate amounts reducing the atmospheric moisture demand, yet may have no immediate effect on existing soil moisture stress in the deeper soil-levels^55^. In addition, in shallow levels, a steady rise in surface temperature facilitates the potential for surface evaporation until it leads to water-stressed conditions, thus limiting the availability of latent heat flux, after which sensible heating systematically increases. This eventually leads to a stronger coupling between soil moisture and surface temperature, facilitating a drought-heatwave cascade. Thus, the CE of both dry-to-hot and hot-to-dry events are relatively more sensitive to changes in PET in the transitional regime. This suggests that the same increase in PET may result in a higher increase in the likelihood (indicated by a greater odd ratio) of such events in this regime. This is possible in arid climates, where scarce vegetation cover during high atmospheric moisture stress driven by a reduction in precipitation leads to an increased albedo from the exposed soil surface, thereby limiting the net radiation during an ongoing drought-heatwave-drought cascade^40^. Thus, a similar increase in VPD and Rn and a decrease in Pr in the arid regime can lead to a relatively higher increase in the likelihood of a dry-to-hot cascade event compared to that in other climate regimes.

## Discussion

Cascading dry and hot events have a significant impact on society^5,6,15,56^. Understanding the causal interactions between these events is very important to better forecast such events and related impacts and requires new methods to account for the measures and scale of such interactions. In this study, we defined a cross-scale interaction-based cascade model framework for measuring the causal effects of global drying on heating and heating on drying. Two sets of distinct event networks, dry-to-hot extreme and hot-to-dry extreme, are constructed using daily root-zone soil moisture and maximum air temperature anomalies. These anomalies are estimated using different combinations of soil moisture and temperature thresholds and by embedding time lags ranging from 1 to 7 days. The CE of drying on heating and vice-versa are subsequently analyzed based on AF^29^, which measures the causal effects in the exposure-to-outcome (dry-to-hot or hot-to-dry) relationship conditioned on multiple confounders (hydroclimatic anomalies).

The results from the study reveal crucial aspects of the causal interactions between dry and hot extreme events, including their global hotspots, hydro-meteorological drivers, and the effect of soil-plant-atmosphere dynamics and background aridity. Extreme to exceptionally strong CEs corresponding to the dry-to-hot and hot-to-dry extreme event cascades occur in a number of hotspot regions, including the lower Mississippi river basin, major parts of the Amazon River basin located in the northern South American continent, central and southern Africa, central and southern Europe, and central, eastern, and southern parts of Asia. Although the hotspot locations for both dry-to-hot and hot-to-dry cascades are similar, the corresponding causal effects vary significantly in their timescale. While the CE for the dry-to-hot extreme event cascades in the hotspot regions is maximum for a time lag of 1 day, that of the hot-to-dry extreme cascades are maximum for a longer time lag, of 2 to 7 days. The longer time-lags associated with the CE in hot-to-dry events are linked to a greater 1-month-lagged autocorrelation of monthly soil moisture anomalies, reflective of the persistency of soil moisture^33,47,57^., The spatial distribution of the odd ratios related to the anomalies of PET, VPD, Pr, and Rn, reveals that the influence of one or more variables shows a dominance on the influence of others. Such interactions are found to vary spatially across the globe, exhibiting a compounding influence on the odds of occurrence of dry-to-hot and hot-to-dry events. VPD and PET exhibit the strongest positive effect on the CE of dry-to-hot events for shorter time lags uniformly across the globe. On the other hand, PET shows a strong positive, and VPD shows a weak positive influence on the CE of hot-to-dry events. The influence of PET and Rn is dominated by the influence of Pr in multiple locations. In the strongly isohydric ecosystem, a negative influence of VPD and PET is observed on the CE of hot-to-dry and dry-to-hot events, respectively. Background aridity seems to have a distinctive control on the CE corresponding to both dry-to-hot and hot-to-dry event cascades across the evaporation regimes for various combinations of selected thresholds (of *RZSM* and *T*_max_) and temporal lags. A relatively stronger CE for dry-to-hot and hot-to-dry extreme event cascade is noted across the semi-arid and sub-humid regimes. Furthermore, the confounding influence of PET, VPD, Pr, and Rn are found to be highly sensitive to changes in aridity and are linked to shifts in energy-partitioning, common in a drought-heatwave-drought cascade.

Soil moisture drying has significant implications on heating^58^, and its impact propagates (cascades) across the physical and human systems affecting agriculture and human health^59,60^. Our study has a broader implication in bridging the gap between disaster risk reduction and climate change adaptation^14,16,17,56^ with the potential to provide a more nuanced framework for assessing interconnected and cascading risks. The results from the study can be usefully transformed to determine the changes in risk of exposure to interconnected hazards^61–63^ and forecasting skill^47,64^. More research is necessary to further extend this framework embedding the influence of large-scale dynamics of weather systems^8,15^, and uncertainties associated with the soil moisture stress in deeper levels in the snow-persistent regions^65^. Our findings can be further channelized to provide a more in-depth understanding of the association of dry and hot cascades with the length of soil-moisture memory^33,47,57^, anticyclonic circulations and blocking^66,67^, land- and vegetation-atmosphere coupling^19,49,50,68^, regional moisture transport^15,69^, vegetation fluxes^70^, water use efficiency^41,45,71^, compound changes in climate variability^72^, and large-scale teleconnections^15^.

## Methods

### Data

In this study, global gridded daily root-zone soil moisture (RZSM) is obtained for the period 1980-2018 from the third version of the Global Land and Evaporation Amsterdam Model (GLEAM v3.3a;^73^) available at https://www.gleam.eu/. Daily total precipitation (Pr), maximum 2 m air temperature (Tmax), VPD, and surface net radiation (Rn) is derived for the period, 1980-2018, from the European Centre for Medium‐Range Weather Forecasts Reanalysis 5 (ERA5; https://cds.climate.copernicus.eu/cdsapp#!/home). Monthly total potential evaporation (PE) data is also obtained for the same period from ERA5 for the calculation of AI^48^. Daily PET data is derived with the Priestley and Taylor (*PT*) evaporation model using surface latent heat flux, surface sensible heat flux, surface pressure data, and daily average temperature data from the ERA5 (see Supplementary Text 1). VPD is calculated using daily dew point temperature, daily mean 2 m air–temperature, and daily surface pressure obtained from the ERA5 data archives (see Supplementary Text 2 for Method). The GLEAM v3.3a combines various satellite-sensor products and ERA5 net radiation, and air temperature to provide relatively more accurate land surface estimates compared to other satellite- and model-based evaporation models^74,75^. While the GLEAM v3.3a dataset is available directly at daily timescale for every 0.25° × 0.25° pixels worldwide, ERA5 provides data at the same spatial resolution but for hourly timesteps. Isohydricity for a given ecosystem is estimated using VOD data obtained from the global LPDR version 3^31^. The LPDR was generated using calibrated microwave brightness temperature records from the Advanced Microwave Scanning Radiometer for EOS (AMSR-E) on the NASA EOS Aqua satellite, and the Advanced Microwave Scanning Radiometer 2 (AMSR2) sensor on the JAXA GCOM-W1 satellite. The VOD data are available at daily multi-frequency, ascending, and descending orbits extending from June 19, 2002, to December 31, 2020. Here, we use the X-band-based VOD data from the ascending orbits at 1:30 AM (referred to as midnight) and the descending orbits at 1:30 PM (referred to as midday)^76^ from the year 2003 to 2018, fairly consistent with our study period, 1980–2018. The methodology used for estimating the isohydricity is provided in Supplementary Text 3.

### Determining cascading dry and hot event network

The cascading hot and dry event network is formalized in two steps, as discussed below.

Estimation of dry and hot events: Dry events are identified using daily RZSM by applying a threshold-based approach. In this study, we use three different thresholds, 1, 5, and 10 percentiles of RZSM, to identify three types of dry events, separately. Specifically, dry events are identified when the daily RZSM falls below the daily climatological (1980–2018 period) threshold of 1, 5, and 10 percentile, which is considered harmful to crop yield (U.S. Dry Monitor (USDM)^77^). In the analysis, we use 99, 95, and 90th percentile thresholds of daily *T*_max_ to identify three types of hot events, separately. Precisely, hot events are defined as events during which the daily Tmax exceeds its daily climatological (99, 95, and 90) percentile threshold for the 1980–2018 period^78,79^. Note that we have used a daily climatological threshold which inherently considers the influence of different climatology of each month or season on the dry/hot indicators. Both dry and hot events were identified for the 1980–2018 period, and the respective daily climatological thresholds were calculated using the whole 39-year time series.

Construction of temporal network: Two types of cascade event networks are constructed in this study to capture the cross-scale interaction^24,27^ between dry and hot event days for the 1980–2018 period. Here, we focus on the dry-to-hot event cascade network to determine the CE of drying on heating, and the hot-to-dry event cascade network to determine the CE of heating on drying. The dynamical associations between the dry and hot event days are evaluated based on lagged time intervals (*T*) embedded in these two temporal networks. Cascading events are generally referred to as the sequential occurrence of events in a dynamical system with 1 day^6^ or multiple time intervals between the occurrences^21,22,56,80^. CEs associated with the dry-to-hot (hot-to-dry) event cascade is defined as the sequential occurrence of a dry (hot) day followed by a hot (dry) day at pre-defined time intervals of 1–7 days. These time intervals are selected for a window of up to 7 days because of the potential increases in difficulty to cope with the socio-ecological impacts of such events as the time window shrinks to a sub-weekly scale^22^.

### Estimation of CEs

The primary objective of the study is to explore the CE of drying on heating and vice-versa, associated with the dry-to-hot and hot-to-dry event networks. For a given network (dry-to-hot or hot-to-dry), the CE is determined based on the causal interaction between drying and heating. In this study, the CE is quantified based on a metric called AF^32^. The AF is a population-specific measure of the proportion of preventable outcomes, e.g., hot (dry) day occurrences, had all days in the time period been unexposed to dry (hot) events. It is a robust technique popularly used in modern epidemiology and public health and can be implemented to measure the exposure-outcome relationship by taking into account necessary confounding measures^81,82^.

In the following sections, we discuss the design of the cascade model, and how it is implemented within a logistic regression framework using a method of regression standardization to measure the causal interactions between drying and heating measured by AF.

Model framework: In a dynamical system, causal interactions can occur through direct or indirect propagation of information within a network consisting of the exposure, outcome, and confounders^25^. In most dynamical systems, the confounders have a causal association with both the outcome and the exposure variable. If confounding (or independent) effects are not accounted for, it may lead to spurious relationships and endogeneity^23^. Consequently, to obtain a robust measure of the effect of the exposure variable on the outcome, it is important to isolate the main effect of the exposure variable on the outcome variable by accounting for all other variables as confounders^29^.

The pathway of information propagation in a dynamical system can be demonstrated by using DAGs. DAGs present a graphical representation of the problem of confounding^25^. Let *Z* denote a set of confounders that control both the outcome and the exposure variable. DAG can describe the confounding by *Z* of the causal relationship between the exposure variable, *X*, and the outcome variable, *Y*, as shown in Fig. 1a. In the case of both dry-to-hot and hot-to-dry event networks, four confounding variables (*z* ⊆ *Z*) are used, such as precipitation (Pr), VPD, surface net radiation (Rn), PET, that are known to have significant control on both dry and hot events^83^.

*Estimation of AF based on logistics regression*: We first identify the dry and hot day occurrences based on the definition of cascading events discussed above. The occurrences and non-occurrences of dry and hot days are subsequently transformed into binary (0/1) time series, such that, an occurrence is denoted by 1 and a non-occurrence is denoted by 0. AF was then calculated by fitting a logistic regression model to the binary exposure, *X*, and the binary outcome, *Y*, with model adjustments specifically made to include the confounders (*Z*). The logit regression is implemented for the selected time intervals (*T* = 1–7) separately. Hereafter in this study, this framework is referred to as *X*_*t*_*Y*_*t+T*_, denoting the causal effect of X at time-step, *t* on Y at time-step, *t* + *T*, being measured for *X* and *Z* lagged by T days. The event-to-event temporal network applied to this framework is illustrated in Fig. 1b.

For binary outcomes and exposure, AF can be defined as in Eq. (1)^32^,

1 $${{{{{\rm{AF}}}}}}=1-\frac{P({Y}_{0}=1)}{P(Y=1)}$$

where *P*(*Y*_0_ = 1) is the counterfactual probability of outcome if the exposure *X* is eliminated (i.e., *X* set to 0), and *P*(*Y* = 1) is the factual probability of an outcome in the population.

In this case, the formulation of AF can be further expanded as

2 $${{{{{\rm{AF}}}}}}=1-\frac{P(Y=1|X=0,\, Z=z)}{P(Y=1)}$$

The AF thus measures the proportion of outcome events (e.g., hot days) that would be prevented if the exposure events (e.g., dry days) were eliminated from the population for predefined levels of confounders, *Z*.

The estimation of AF is carried out in four steps.

*Step 1*. A regression model is fitted to the observed data. Unlike the linear, and log-linear models, logistic regression is a standard choice for estimating AF due to its ability to yield probabilities between 0 and 1. The logistic regression model is defined as

3 $${{{{{\rm{log }}}}}}{it}\left\{\Pr \left(Y=1{{{{{\rm{|}}}}}}X=0,\, Z\right)\right\}=g\left(X,\, Z{{{{{\rm{;}}}}}}\beta \right)$$

Here *g*(.) is an additive function of the variables *X* and Z and could be specified as ${\beta }_{0}+{\beta }_{1}X+{\beta }_{2}Z$, where *β* is the parameter vector of the logit model.

*Step 2*. The fitted model is used to estimate $\Pr \left(Y=1{|X}=x,\, Z\right)$ for the fixed level of *X* = *x* (here, *x* = 0) and for each observed level of *Z* in the dataset.

*Step 3*. A regression standardization^30^ is then implemented to the fitted model to estimate marginal measures of association. This method uses the logistic regression model to estimate the risk ratios of the outcome (*Y*), for *X* = 0 at every pre-defined level of the measured confounders, *Z*. These estimates are averaged over the sampling distribution of *Z* to produce a standardized risk, for *X* = 0. Thus, if *Z* is sufficient for cofounding control, then

4 $$P\left({Y}_{0}=1\right)=E\left[P\left(Y=1{{{{{\rm{|}}}}}}X=x,\, Z\right)\right],$$

5 $$\hat{p}({Y}_{0}=1)=\frac{\mathop{\sum }\limits_{i=1}^{n}\hat{p}(Y=1|X=0,\, {Z}_{i})}{n},$$

where *Z*_*i*_ is the observed level of *Z* for the observation, *i*, *i* = 1, …., *n*, and $\hat{p}(Y=1|X=0,\, {Z}_{i})$ is the estimate of $p(Y=1|X=0,\, {Z}_{i})$ obtained from the fitted regression model.

*Step 4*. Once, *P*(*Y*_0_ = 1) is estimated following steps 1–3, it is directly implemented in Eq. 1 to calculate AF.

It is important to note that AF is estimated for each model framework (*XY*_*T*_) separately and denoted by AF_*T*_.

Estimation of confidence intervals: We construct a standard Wald-type 95% confidence interval for the desired effect measure based on the delta method^84^.

Let *p* be the vector of counterfactual probabilities with an estimate, $\hat{p}$, and let g(*p*) be the desired effect of measure with an estimated effect, $g(\hat{p})$. Therefore, $g(\hat{p})$can be shown to have an asymptotic normal distribution, with variance given as,

6 $${{{{\mathrm{var}}}}}\{g(\hat{p})\}=\frac{\partial g(p)}{\partial p}{{{{\mathrm{var}}}}}(\hat{p})\frac{\partial g(p)}{\partial {p}^{T}}.$$

An estimate of the variance, ${{\mbox{va}}}\hat{r}\{g(\hat{p})\}$is calculated by replacing *p* and ${{{{\mathrm{var}}}}}(\hat{p})$ in Eq. (6) by $\hat{p}$, and ${{\mbox{va}}}\hat{r}(\hat{p})$.

Finally, the estimated variance is used to construct a standard Wald-type 95% confidence interval for *g*(*p*)given as

7 $${{{{{\rm{CI}}}}}}=g(\hat{p})\pm 1.96\sqrt{{{\mbox{va}}}\hat{r}\{g(\hat{p})\}}$$

Subsequently, the null hypothesis that exposure has no effect on the outcome is rejected when all values in the confidence interval fall on the same side of zero (either all positive or all negative).

## Supplementary information

Supplementary Information

## Data Availability

The data sets analyzed during the current study are available at Global Land and Evaporation Amsterdam Model (GLEAM v3.3a; https://www.gleam.eu/), European Centre for Medium‐Range Weather Forecasts Reanalysis 5 (ERA5; https://cds.climate.copernicus.eu/cdsapp#!/home), and National Snow and Ice Data Center (NSIDC; https://nsidc.org/data/nsidc-0451/versions/3)

## Code availability

Available upon request to the authors.
