# Supplementary material for: Interaction between dry and hot extremes at a global scale using a cascade modeling framework
Source: Nat Commun. 2023 Jan 17;14:277. doi: 10.1038/s41467-022-35748-7 (PMC9845298; doi:10.1038/s41467-022-35748-7)
Supplement: Supplementary file 1 — Supplementary Information [file 41467_2022_35748_MOESM1_ESM.docx]

**Supplementary Information for**

**Interaction between Dry and Hot Extremes at a Global Scale using A Cascade Modeling Framework**

### Sourav Mukherjee^1^, Ashok Kumar Mishra^1^, Jakob Zscheischler^2^, Dara Entekhabi^3^

^1^Glenn Department of Civil Engineering,

Clemson University, South Carolina, USA.

^2^Department of Computational Hydrosystems,

Helmholtz Centre for Environmental Research - UFZ, Leipzig, Germany.

^3^Parsons Laboratory, Department of Civil and Environmental Engineering, Massachusetts Institute of Technology, Cambridge, MA, 02139, USA

Corresponding author: Ashok Kumar Mishra ([ashokm@g.clemson.edu)](mailto:ashokm@g.clemson.edu))

**Contents:**

Text: Text 1-3

Figures: Fig 1 to Fig 21

**Supplementary Text**

**Supplementary Text 1. Calculation of Potential Evapotranspiration using Priestley-Taylor Equation**

The Priestly-Taylor (PT) equation^1^ is extensively used in the calculation of potential evapotranspiration (PET), applied to drought quantification. It is a simplified version of the Penman-Monteith method, where the vapor pressure deficit and convection terms are reduced to an empirical constant, $\alpha(=1.26)$. The PT equation is given as,

$PET=\alpha\times\frac{\Delta\times\left(SHF+LHF\right)}{\lambda_{v}\times\left(\Delta+\gamma\right)}\times1000,$ (1)

where, PET is in mm/day, $\lambda_{v}$ is volumetric latent heat of vaporization, 2453 MJ m^-3^, $\gamma(=0.000665\times P)$is the psychrometric constant (kPa ºC^-1^), LHF is the surface latent heat flux, and SHF is the surface sensible heat flux. Δ is slope of the saturation vapor pressure-temperature curve (kPa ºC^-1^) calculated as,

$\Delta=\frac{4098\times e_{s}}{\left(T_{a}+237.3\right)^{2}}$ (2)

where, *e_s_* is the saturation vapor pressure (kPa) given as,

$e_{s}=6.112\times\left(1+7\times10^{-4}+3.46\times10^{-6}\times P_{surf}\right)\times e^{\frac{17.67T_{a}}{T_{a}+243.5}}$ (3),

*T_a_* is the daily mean 2m air temperature (ºC), and *P_surf_* is the surface pressure (hPa).

**Supplementary Text 2. Estimation of Vapor Pressure Deficit**

In this study, daily vapor pressure deficit (VPD) is calculated by using daily mean dew point temperature, daily mean 2m air temperature, and surface pressure obtained from the ERA5 dataset. The VPD estimation based on these variables is given as^2^,

$AVP=6.112\times f_{w}\times e^{\frac{17.67T_{d}}{T_{d}+243.5}}$ (4)

where, AVP is the actual vapor pressure and T_d_ is the dew point temperature (ºC),

$SVP=6.112\times f_{w}\times e^{\frac{17.67T_{a}}{T_{a}+243.5}}$ (5)

where, SVP is the saturation vapor pressure (kPa), and T_a_ is the daily mean 2m air temperature (ºC), and *f_w_* is given as,

$f_{w}=1+7\times10^{-4}+3.46\times10^{-6}\times P_{surf}$ (6)

where, *P_surf_* is the surface pressure in (hPa).

Finally, VPD is calculated as,

$VPD=AVP-SVP$ (7)

**Supplementary Text 3. Estimation of Ecosystem scale Isohydricity from VOD data**

Under steady state condition, isohydricity is conceptualized as a measure of the relative sensitivity of the transpiration rate and plant hydraulic conductance to declining water availability^3^. Isohydricty can be quantified by the slope of linear regression between leaf water potential and soil water potential given as^3^,

(8)

where Ψ_L_ is the leaf water potential, Ψ_S_ is the root-zone soil water potential, and *σ* and Λ are the slope and intercept of the regression, respectively. A perfectly isohydric species has *σ* close to 0, whereas a perfectly anisohydric species has *σ* close to 1 or >1. Thus, *σ* incorporates the effects of both stomatal regulations by water stress and loss of xylem conductance with reduced soil water^4^. The methodology used for calculating the isohydricity, in this study, is adopted from ^5^. This methodology assumes that xylem-refilling (e.g., the nighttime continuation of sap flux) is nearly complete by 1:30AM local time, during which the Ψ_L_ is restored to values close to Ψ_S_. The main advantage of this assumption is that VOD observations are available during this time. At this stage, it is commonly assumed that Ψ_L_ ≈ Ψ_S_ ^6^, leading to the simplified form of equation 8, given as ^5^,

(9)

Moreover, a linearly proportional relationship exists between the VOD and vegetation water content (VWC; Kg m^-2^):

$VOD=b\times VWC$ (10)

where *b* is a scaling parameter that whose value dependes on the plant structure but it is generally assumed to be constant in time^5,7^.

Since VWC is usually exponentially related to Ψ_L_, it can be further approximated as^5^,

(11)

Substituting Equation (11) in Equation (9) and further simplification leads to^5^,

(12)

Thus, *σ* is the slope of the regression between midnight and midday vegetation optical depth. In this study, global geographic variations in ecosystem isohydricity are assessed assuming a constant value of σ for each grid-point location. It is important to note that σ is calculated using the VOD data for no-precipitation days to avoid contamination from intercepted water in the canopy layers^5^.

**Supplementary Figures**

**
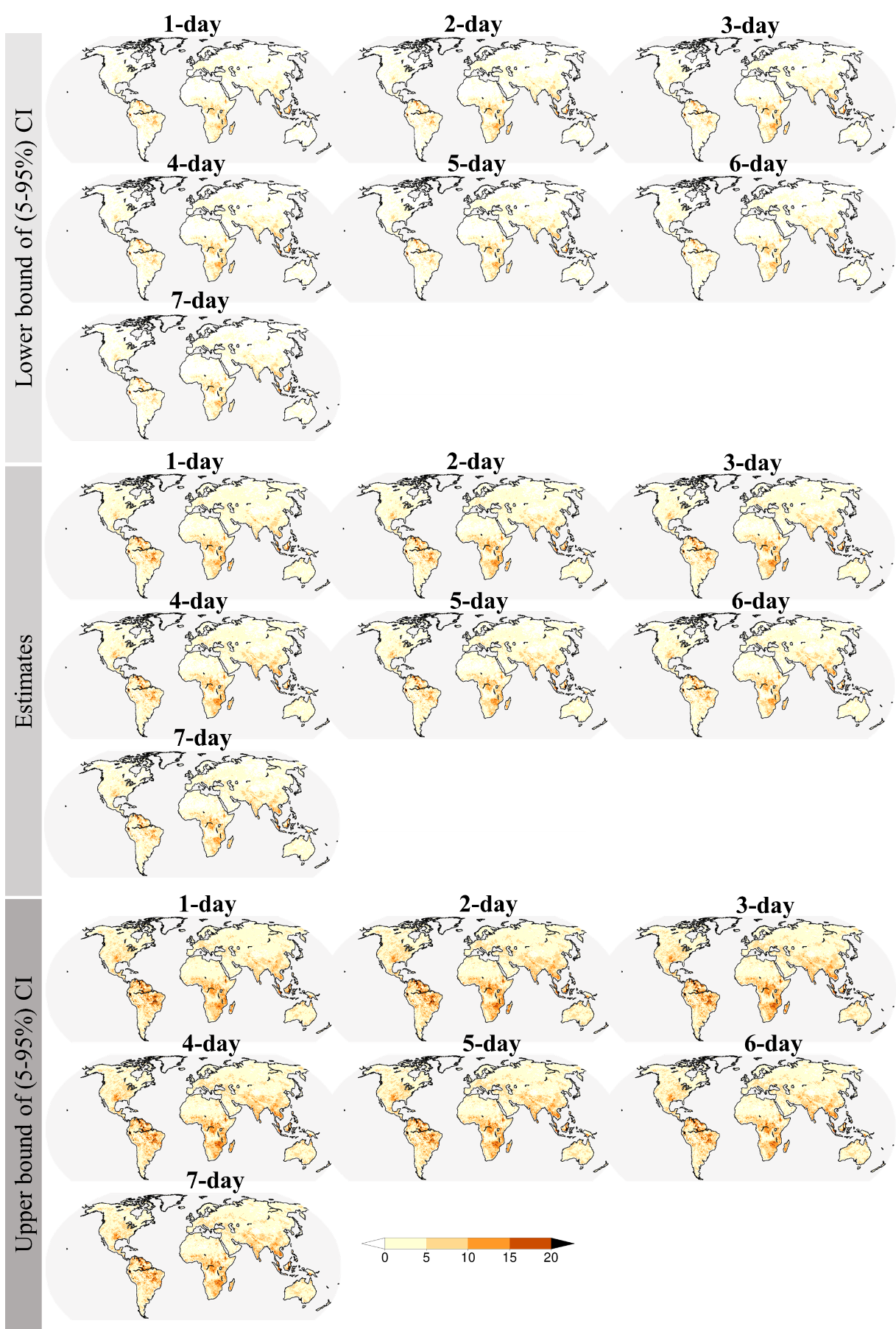
**

**Supplementary Figure 1** Estimates of attribution fraction (AF): spatial maps showing the lower bound, estimates, and upper bound for 5-95% confidence interval of AF (%) corresponding to type of dry-to-hot event cascade, Drought-Heatwave cascade derived using 1 percentile of root-zone-soil-moisture (RZSM), and 99^th^ percentile of daily maximum 2m air temperature (Tmax) as threshold (D1p-H99p; see Methods), considering 0 to 7-days lagged time-intervals.

**
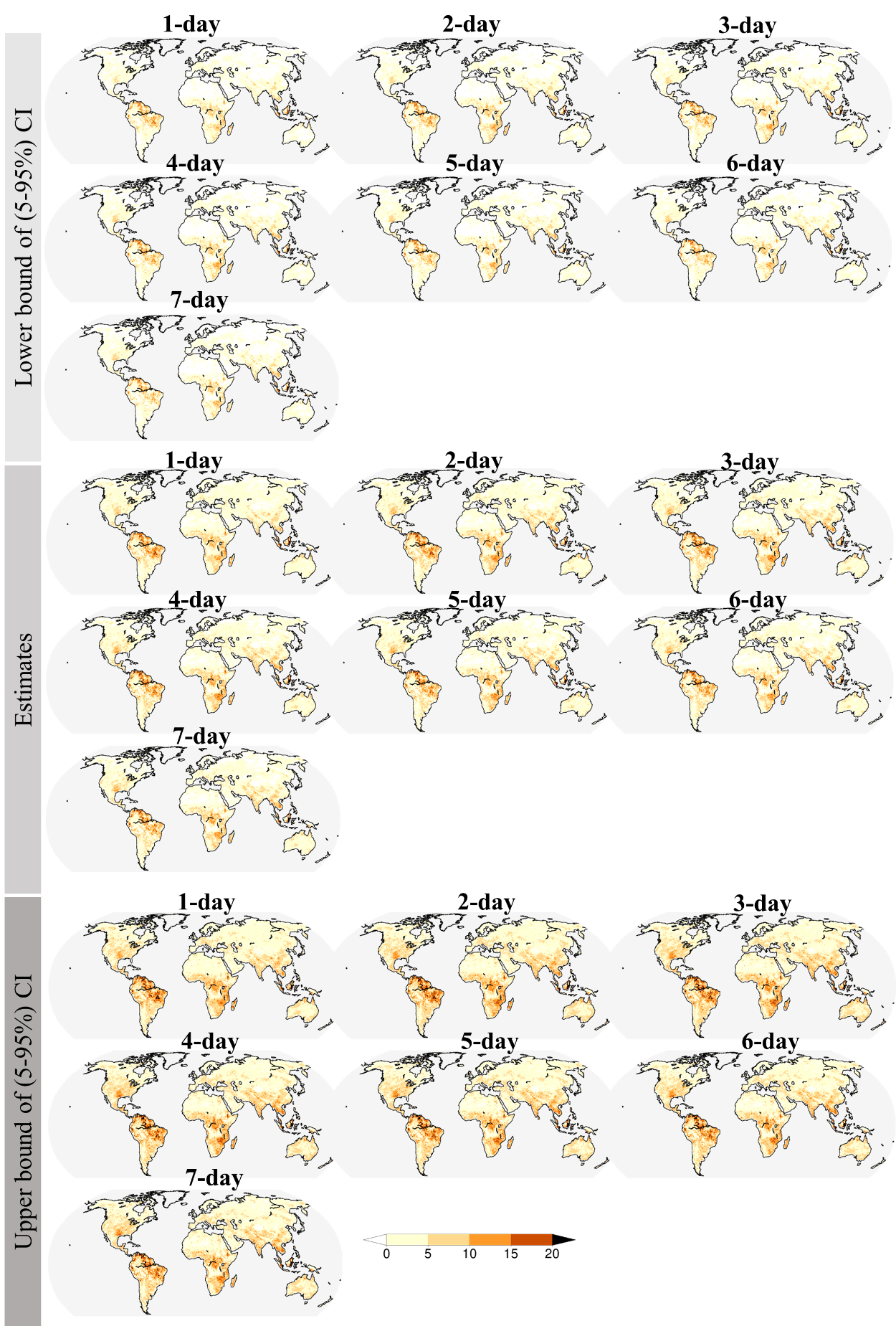
**

**Supplementary Figure 2** Estimates of attribution fraction (AF): same as in Figure 1 but corresponding to Drought-Heatwave cascade derived using 5^th^ percentile of root-zone-soil-moisture (RZSM), and 95^th^ percentile of daily maximum 2m air temperature (Tmax) as threshold (D5p-H95p) event cascade.

**
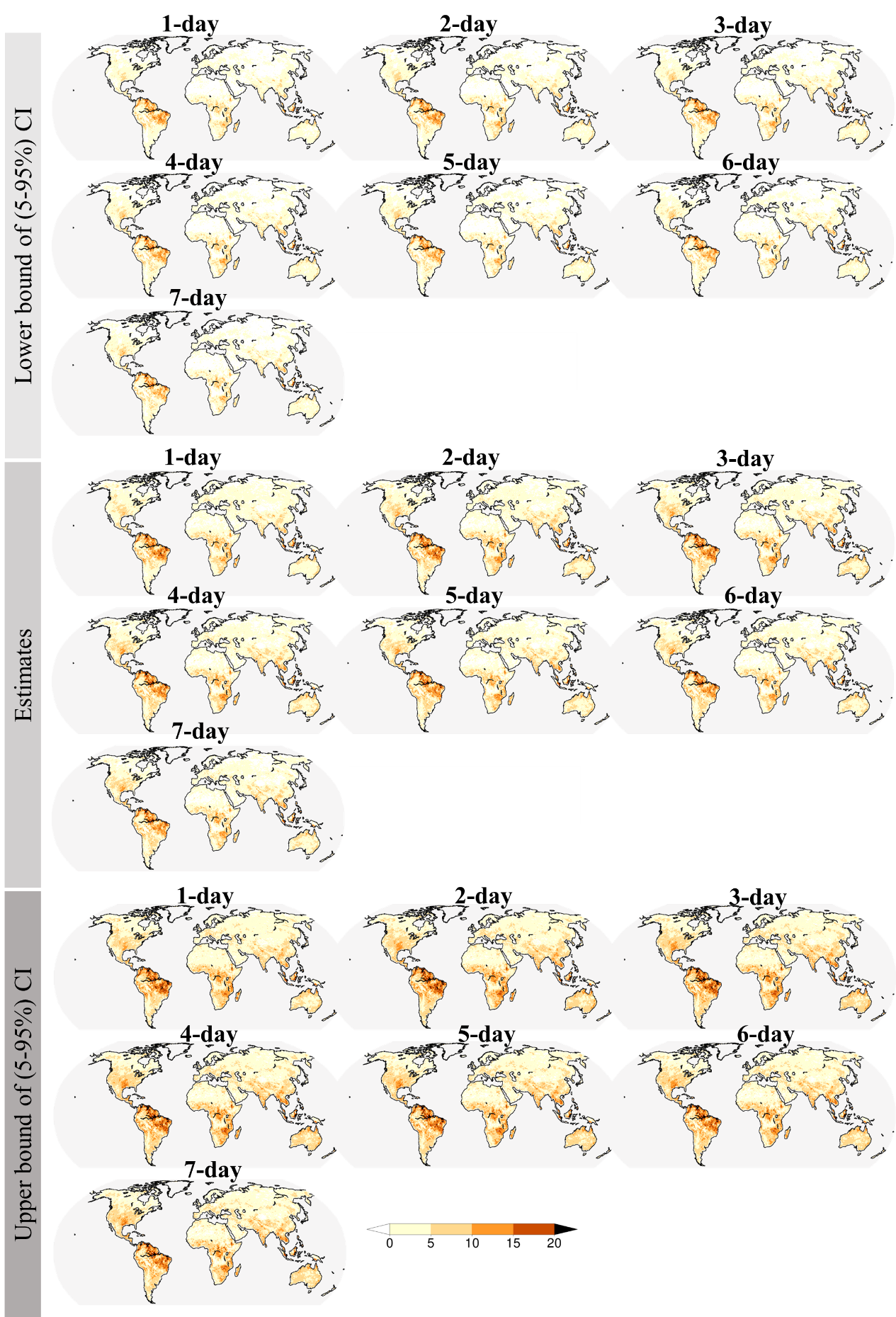
**

**Supplementary Figure 3** Estimates of attribution fraction (AF): same as in Figure 1 but corresponding to Drought-Heatwave cascade derived using 10^th^ percentile of root-zone-soil-moisture (RZSM), and 90^th^ percentile of daily maximum 2m air temperature (Tmax) as threshold (D10p-H90p) event cascade.

**
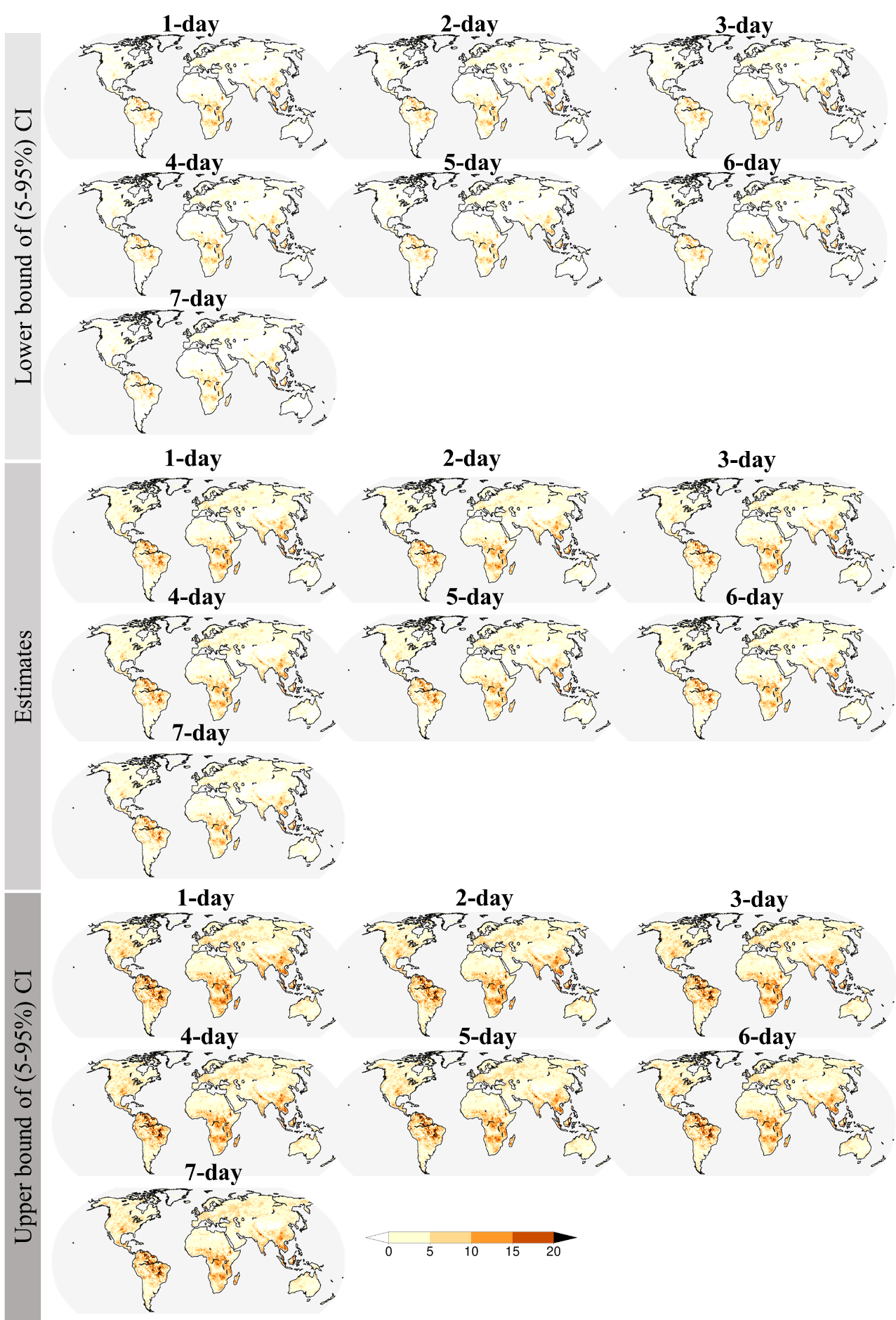
**

**Supplementary Figure 4** Estimates of attribution fraction (AF): same as in Figure 1 but corresponding to Heatwave-Drought cascade derived using 1 percentile of root-zone-soil-moisture (RZSM), and 99^th^ percentile of daily maximum 2m air temperature (Tmax) as threshold (H99p-D1p) event cascade.

**
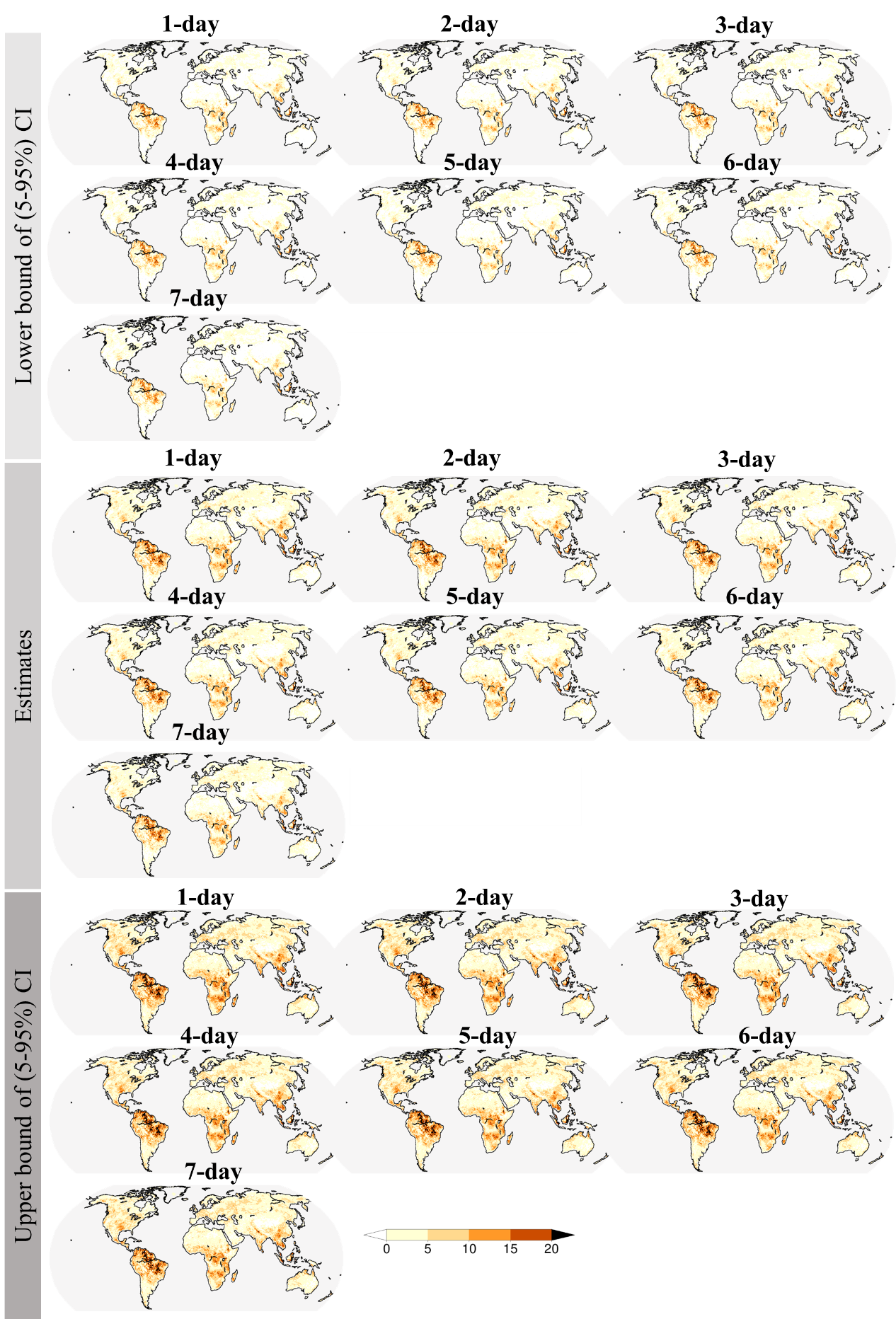
**

**Supplementary Figure 5** Estimates of attributable fraction (AF): same as in Figure 1 but corresponding to Heatwave- Drought cascade derived using 5^th^ percentile of root-zone-soil-moisture (RZSM), and 95^th^ percentile of daily maximum 2m air temperature (Tmax) as threshold (H95p-D5p) event cascade.

**
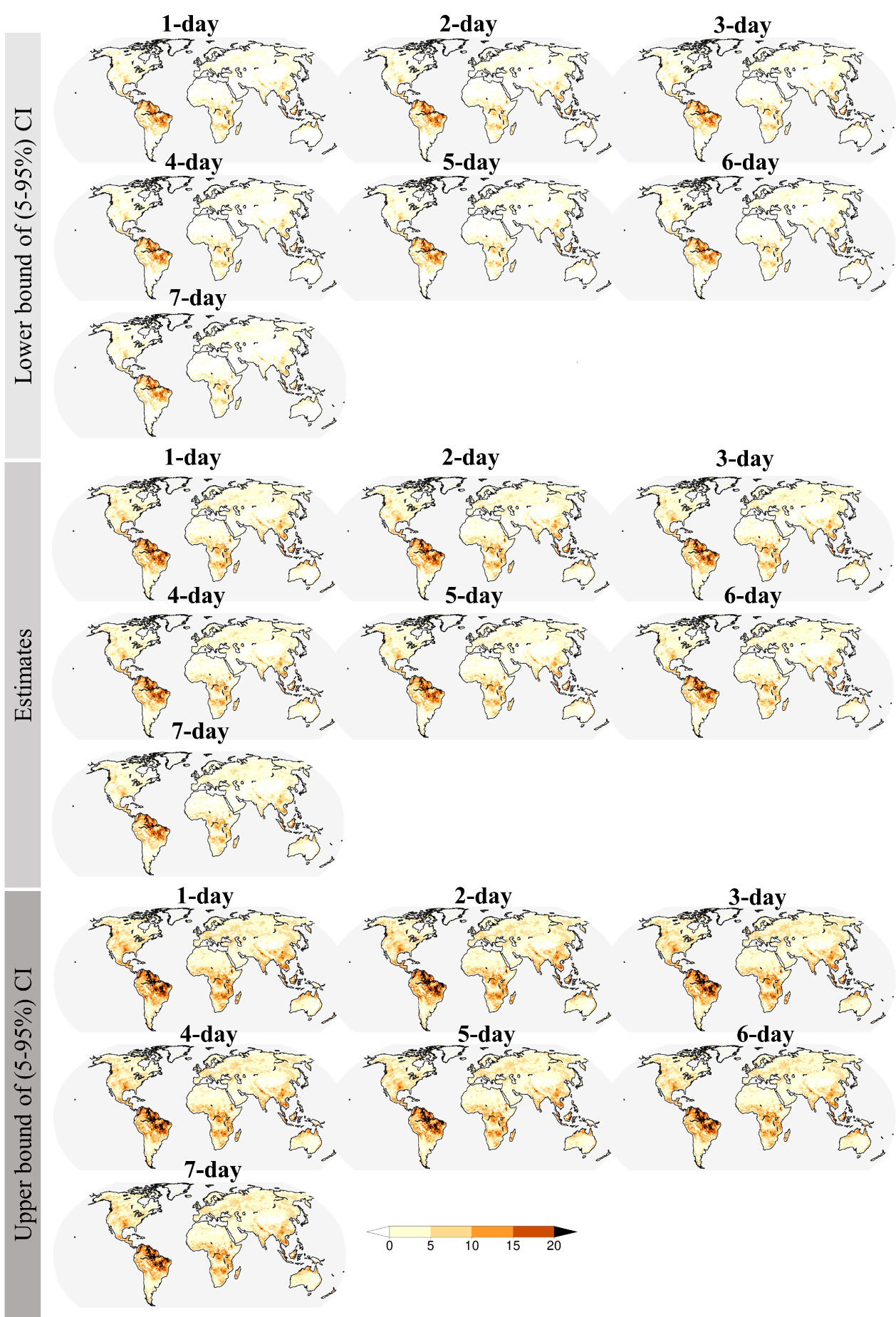
**

**Supplementary Figure 6** Estimates of attributable fraction (AF): same as in Figure 1 but corresponding to Heatwave-Drought cascade derived using 10^th^ percentile of root-zone-soil-moisture (RZSM), and 90^th^ percentile of daily maximum 2m air temperature (Tmax) as threshold (H90p-D10p) event cascade.


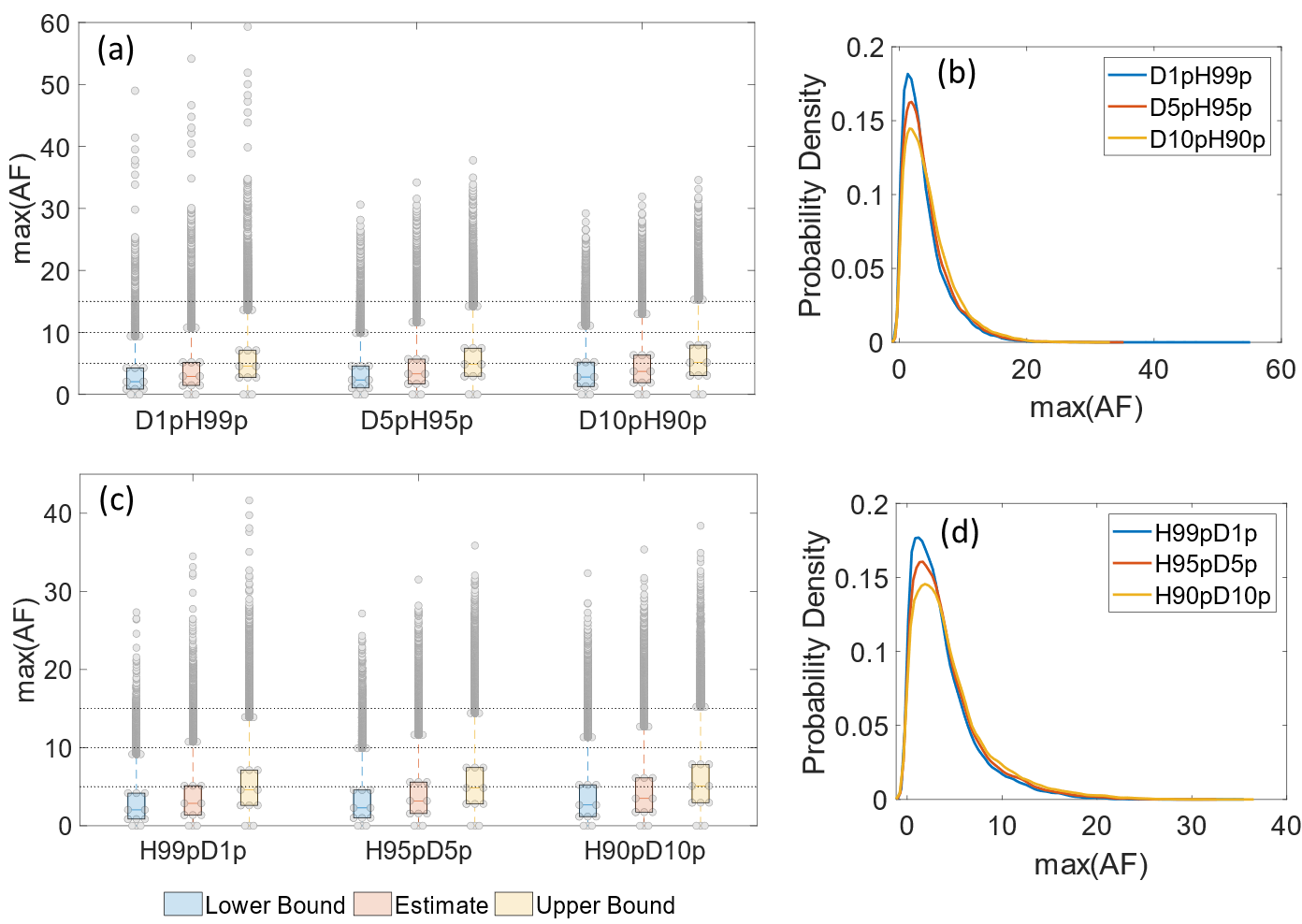


**Supplementary** **Figure 7** (a) Estimates of attribution fraction (AF): boxplots showing the spatial distribution of maximum AF_T_ (%) estimates (in red) and the lower (in blue) and upper (in yellow) 2.5-97.5% confidence intervals for the dry-to-hot event for T=1,2,3,4,5,6, and 7-days, (b) probability density estimates of maximum AF_T_ (%) for the dry-to-hot event cascades, (c-d) same as in (a-b) but for hot-to-dry event cascades.


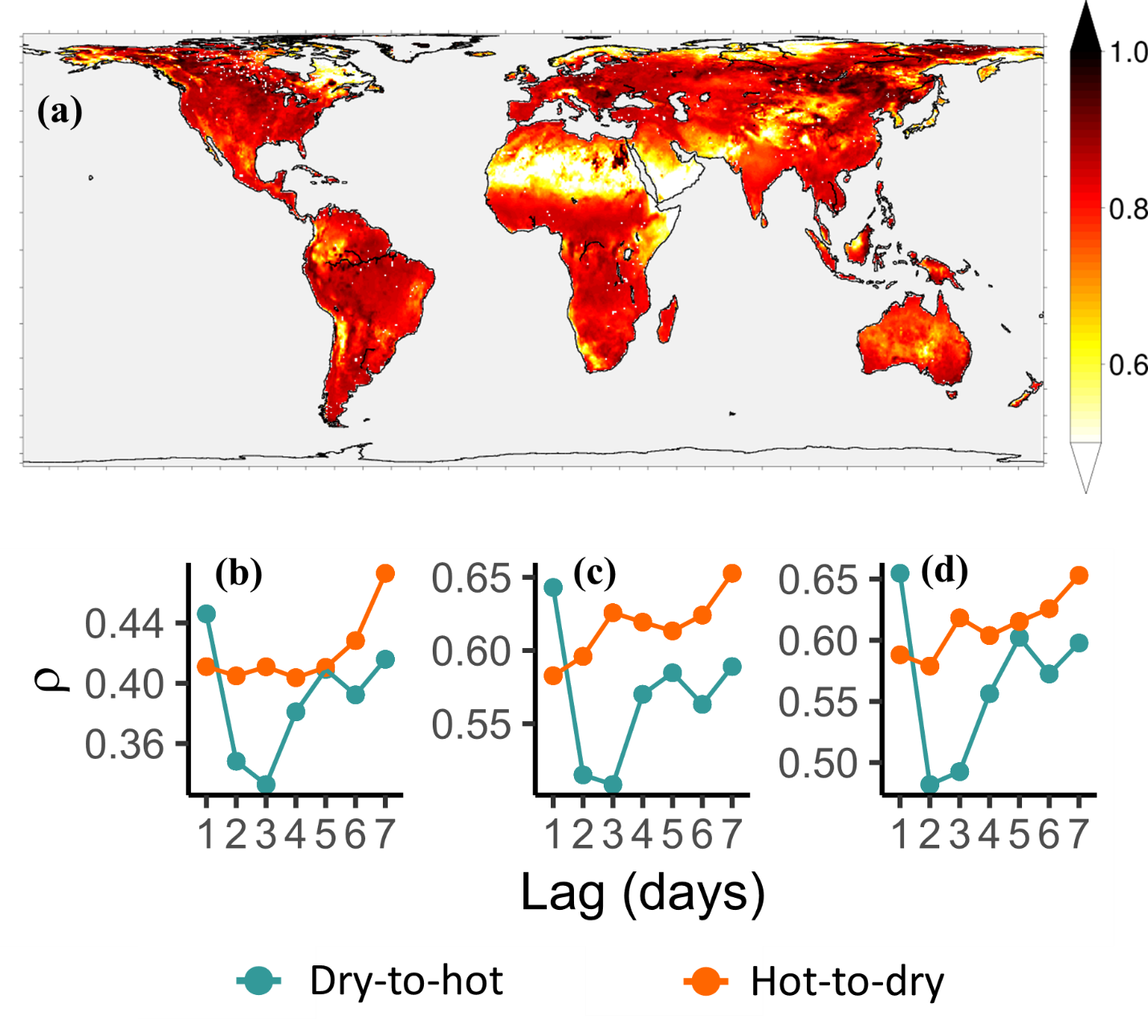


**Supplementary Figure 8** Association with root-zone-soil moisture (RZSM) memory (a) Spatial distribution of one-month lagged autocorrelation coefficient (ρ, derived based on Pearson’s rank correlation method) of monthly RZSM anomalies, (b-d) grid-point average of the autocorrelation coefficients for the temporal lags of maximum CEs associated with (b) dry-to-hot (D1pH99p), and hot-to-dry (H99pD1p) with 1 percentile of RZSM, and 99^th^ percentile of daily maximum 2m air temperature (Tmax) as threshold, similarly with (c) D5pH95p, and H95pD5p, and (d) D10pH90p, and H90pD10p event cascade.

**
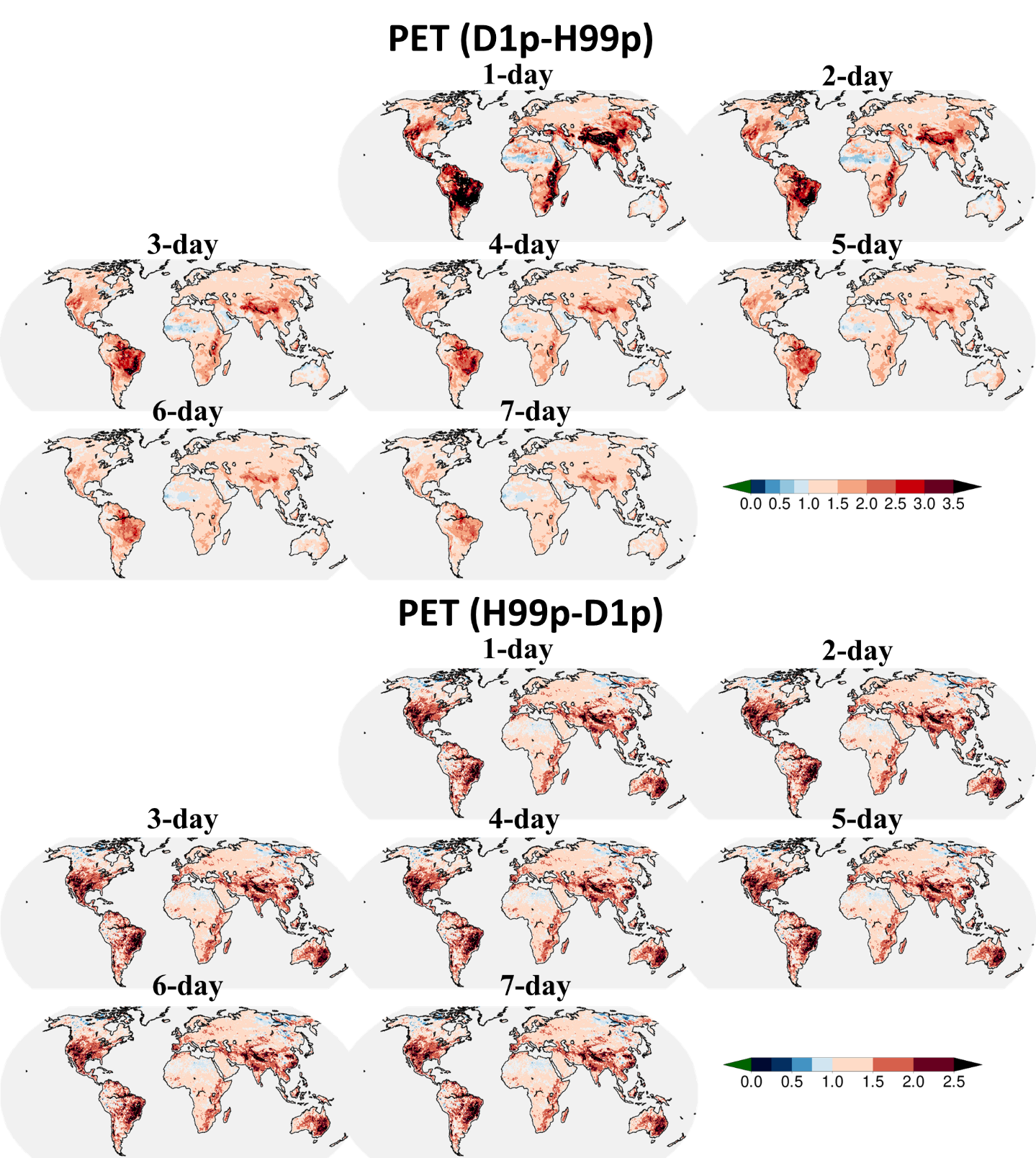
**

**Supplementary** **Figure 9** Role of confounders: Spatial distribution of statistically significant (at 5% significance level) odd ratios (*exp(β)*) corresponding to potential evapotranspiration (PET) anomalies calculated by fitting the logistic regression model for the dry-to-hot (D1p-H99p: top panel) and hot-to-dry (H99p-D1p: bottom panel) event cascade with 1 percentile of root-zone-soil-moisture (RZSM), and 99^th^ percentile of daily maximum 2m air temperature (Tmax) as threshold for a lag of 1 to 7 days.

**
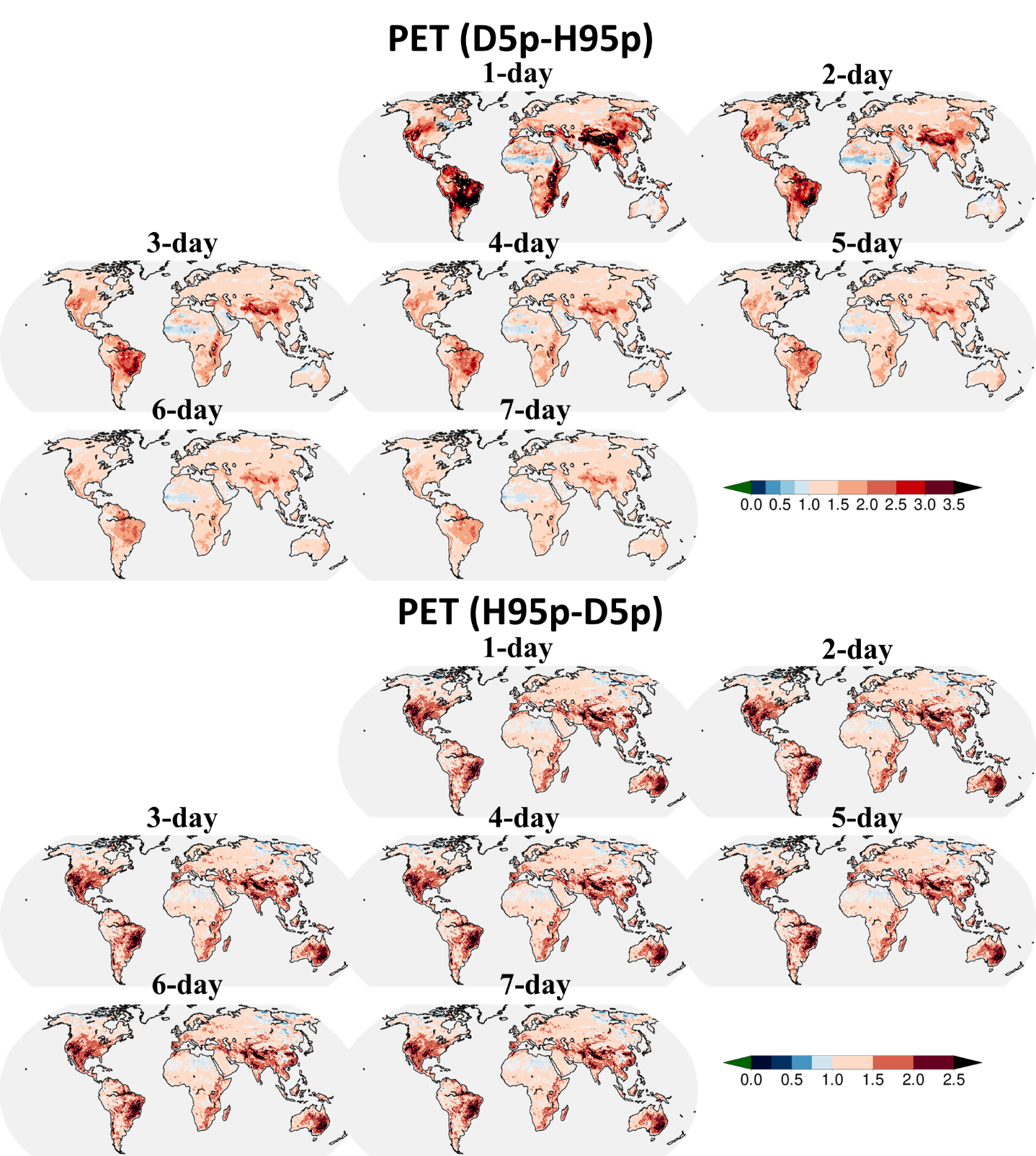
**

**Supplementary** **Figure 10** Role of confounders: same as in Figure 9 but for the dry-to-hot (D5p-H95p: top panel) and hot-to-dry (H95p-D5p : bottom panel) event cascade with 5^th^ percentile of root-zone-soil-moisture (RZSM), and 95^th^ percentile of daily maximum 2m air temperature (Tmax) as threshold.

**
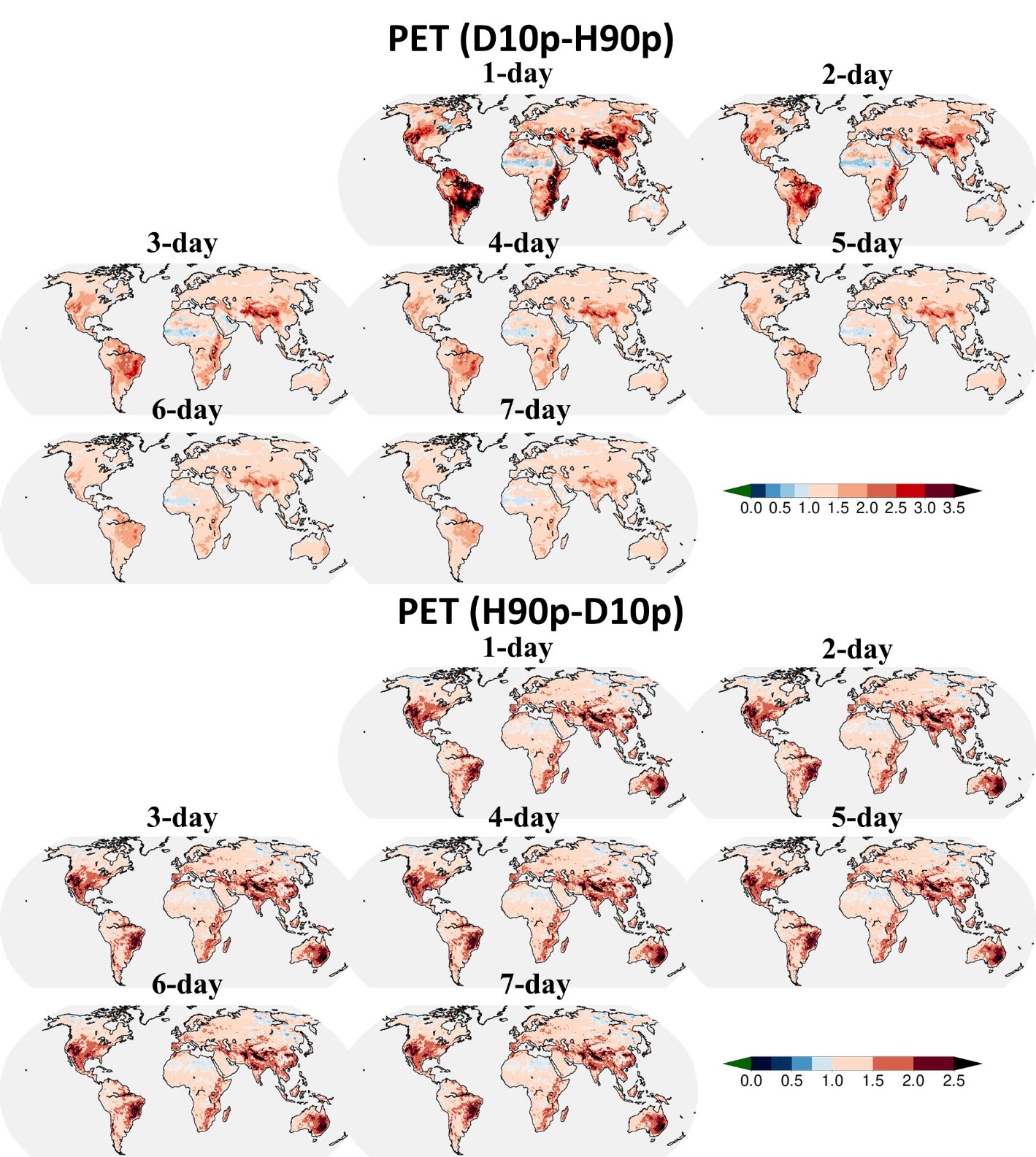
 Supplementary Figure 11** Role of confounders: same as in Figure 9 but for the dry-to-hot (D10p-H90p: top panel) and hot-to-dry (H90p-D10p: bottom panel) event cascade with 10^th^ percentile of root-zone-soil-moisture (RZSM), and 90^th^ percentile of daily maximum 2m air temperature (Tmax) as threshold.

**
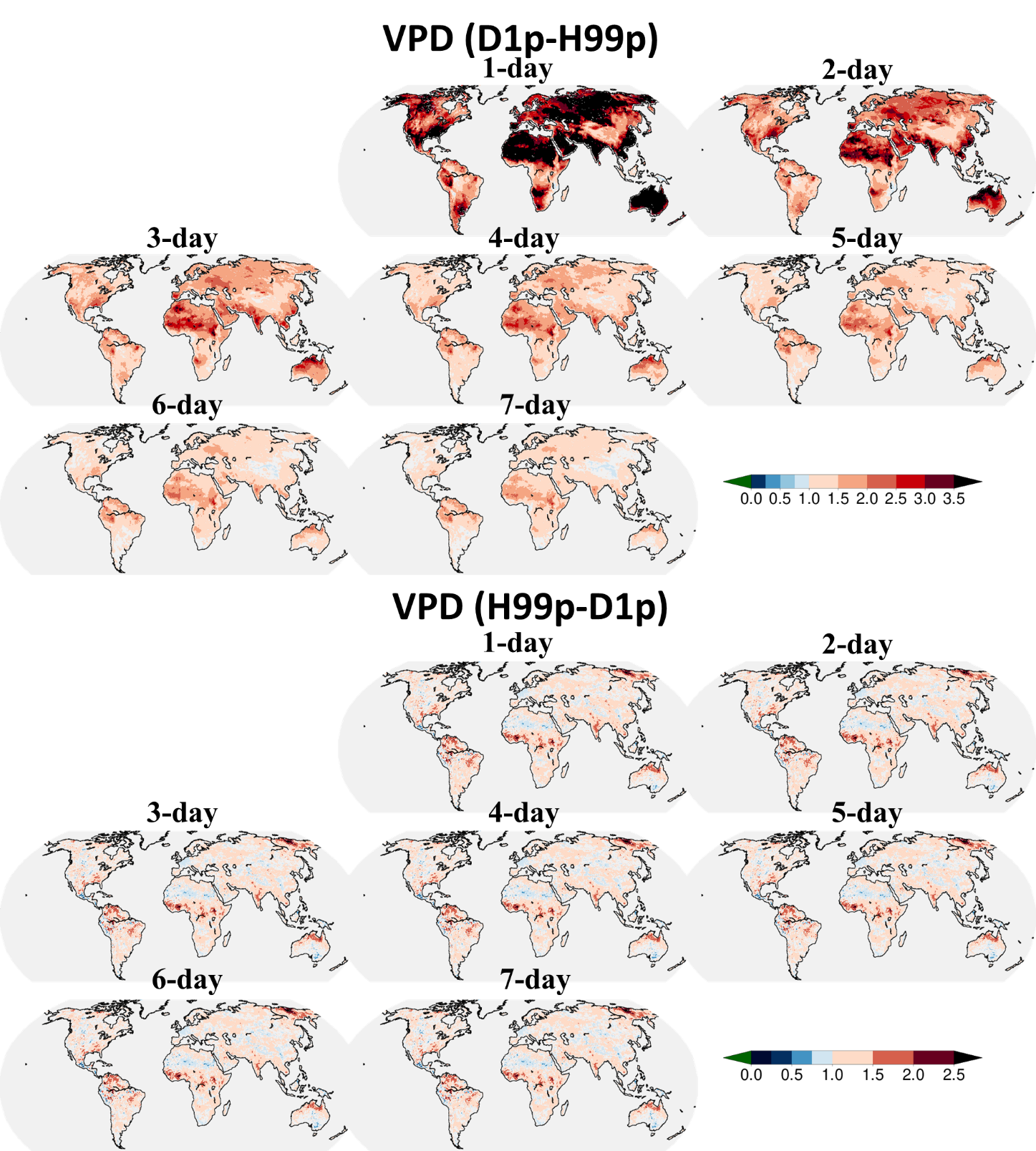
 Supplementary Figure 12** Role of confounders: spatial distribution of statistically significant (at 5% significance level) odd ratios (*exp(β)*) corresponding to vapor pressure deficit (VPD) anomalies calculated by fitting the logistic regression model the dry-to-hot (D1p-H99p: top panel) and hot-to-dry (H99p-D1p: bottom panel) event cascade with 1 percentile of root-zone-soil-moisture (RZSM), and 99^th^ percentile of daily maximum 2m air temperature (Tmax) as threshold for a lag of 1 to 7 days.

**
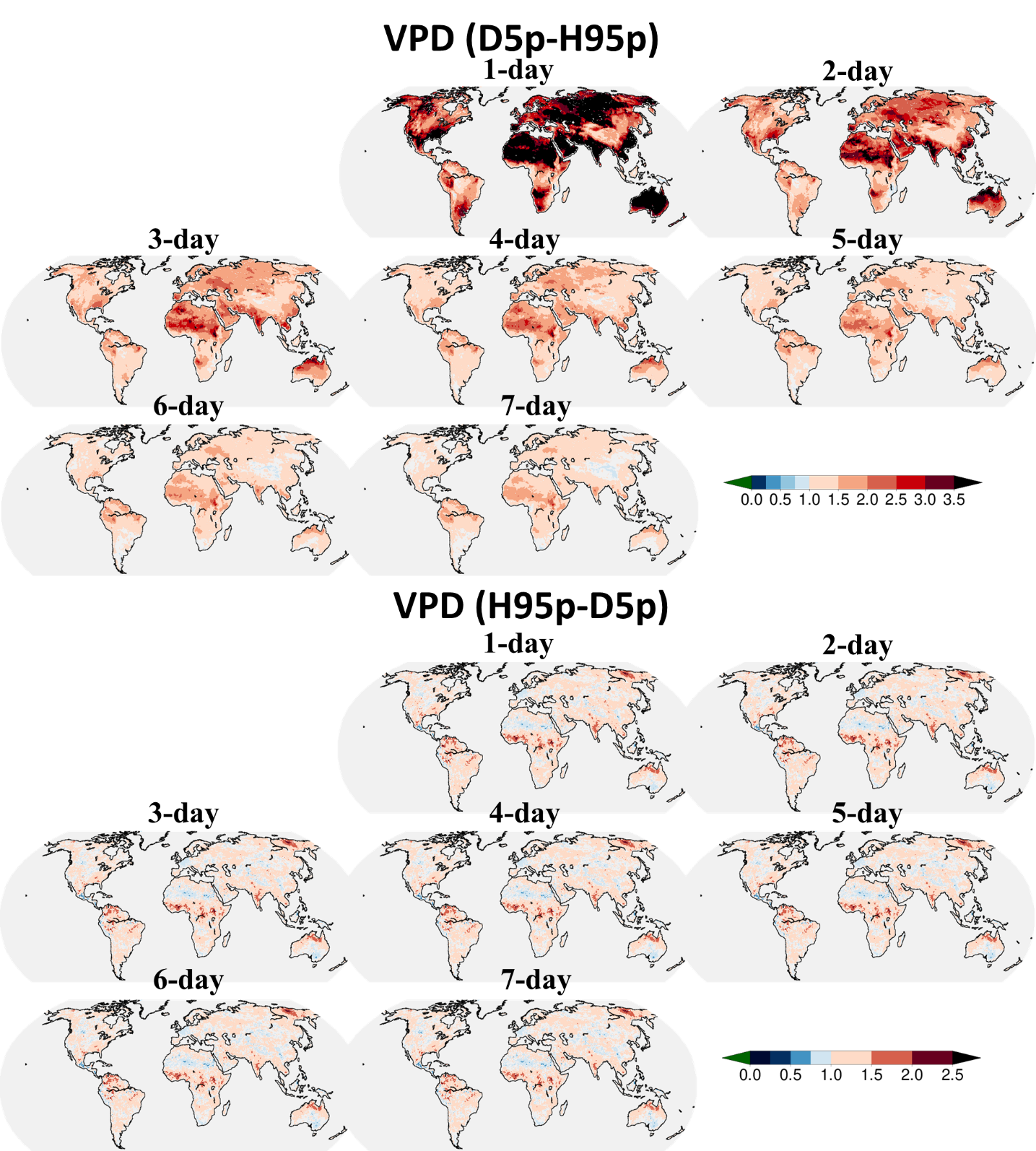
 Supplementary Figure 13** Role of confounders: same as in Figure 12 but for the dry-to-hot (D5p-H95p: top panel) and hot-to-dry (H95p-D5p: bottom panel) event cascade with 5^th^ percentile of root-zone-soil-moisture (RZSM), and 95^th^ percentile of daily maximum 2m air temperature (Tmax) as threshold. **
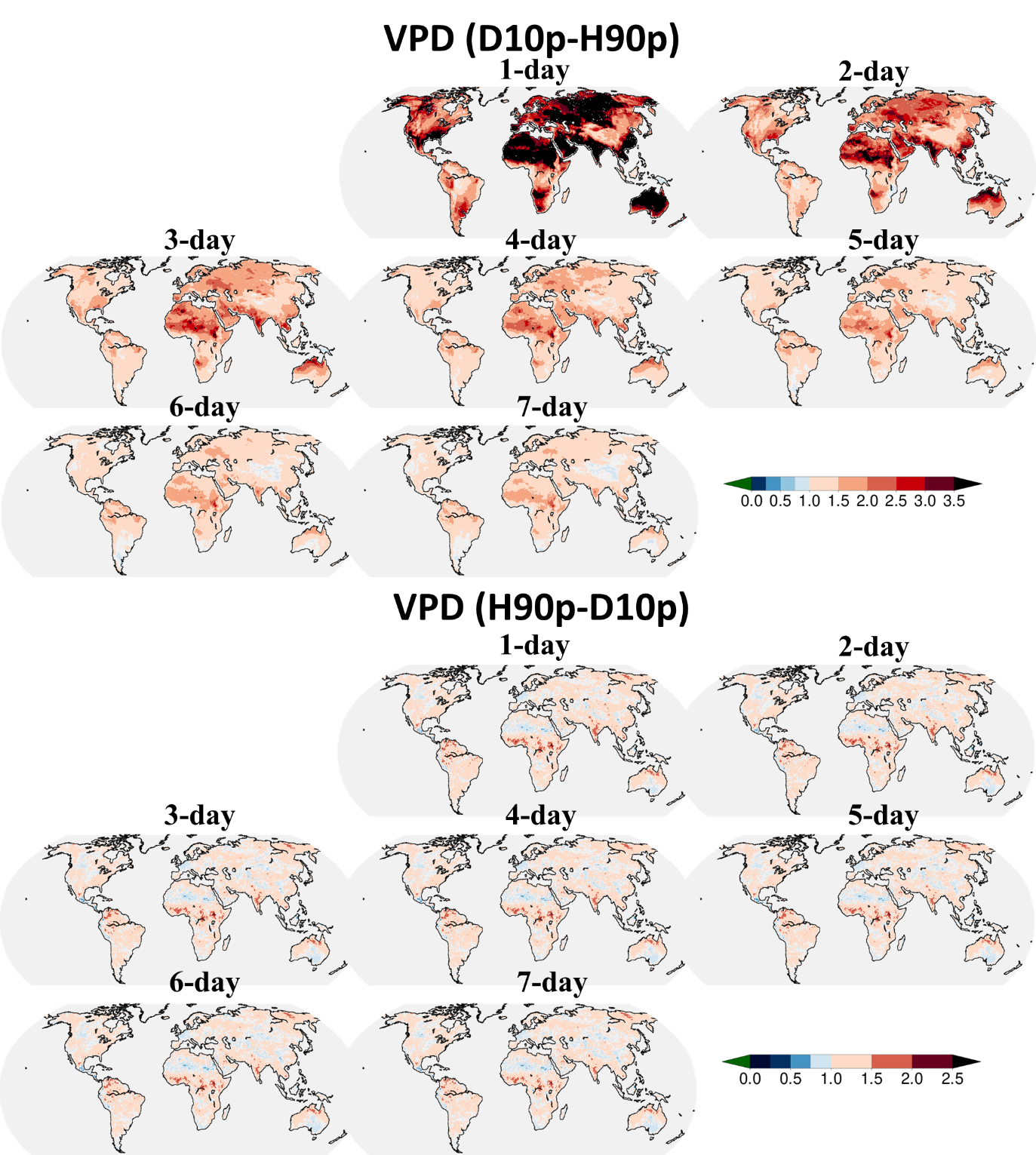
 Supplementary Figure 14** Role of confounders: same as in Figure 12 but for the dry-to-hot (D10p-H90p: top panel) and hot-to-dry (H90p-D10p: bottom panel) event cascade with 10^th^ percentile of root-zone-soil-moisture (RZSM), and 90^th^ percentile of daily maximum 2m air temperature (Tmax) as threshold.

**
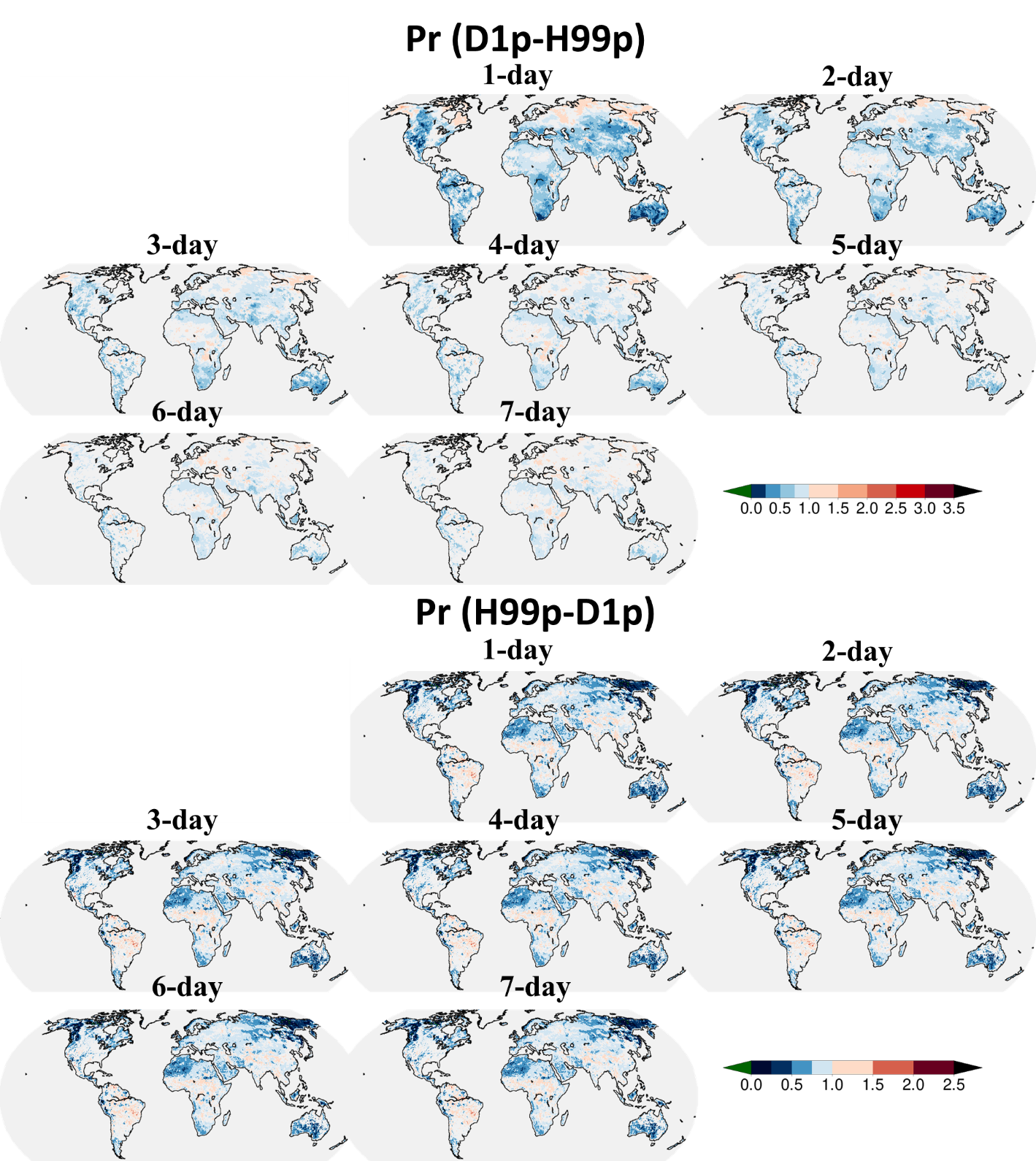
**

**Supplementary Figure 15** Role of confounders: spatial distribution of statistically significant (at 5% significance level) odd ratios (*exp(β)*) corresponding to precipitation (Pr) anomalies calculated by fitting the logistic regression model for the dry-to-hot (D1p-H99p: top panel) and hot-to-dry (H99p-D1p: bottom panel) event cascade with 1 percentile of root-zone-soil-moisture (RZSM), and 99^th^ percentile of daily maximum 2m air temperature (Tmax) as threshold for a lag of 1 to 7 days.

**
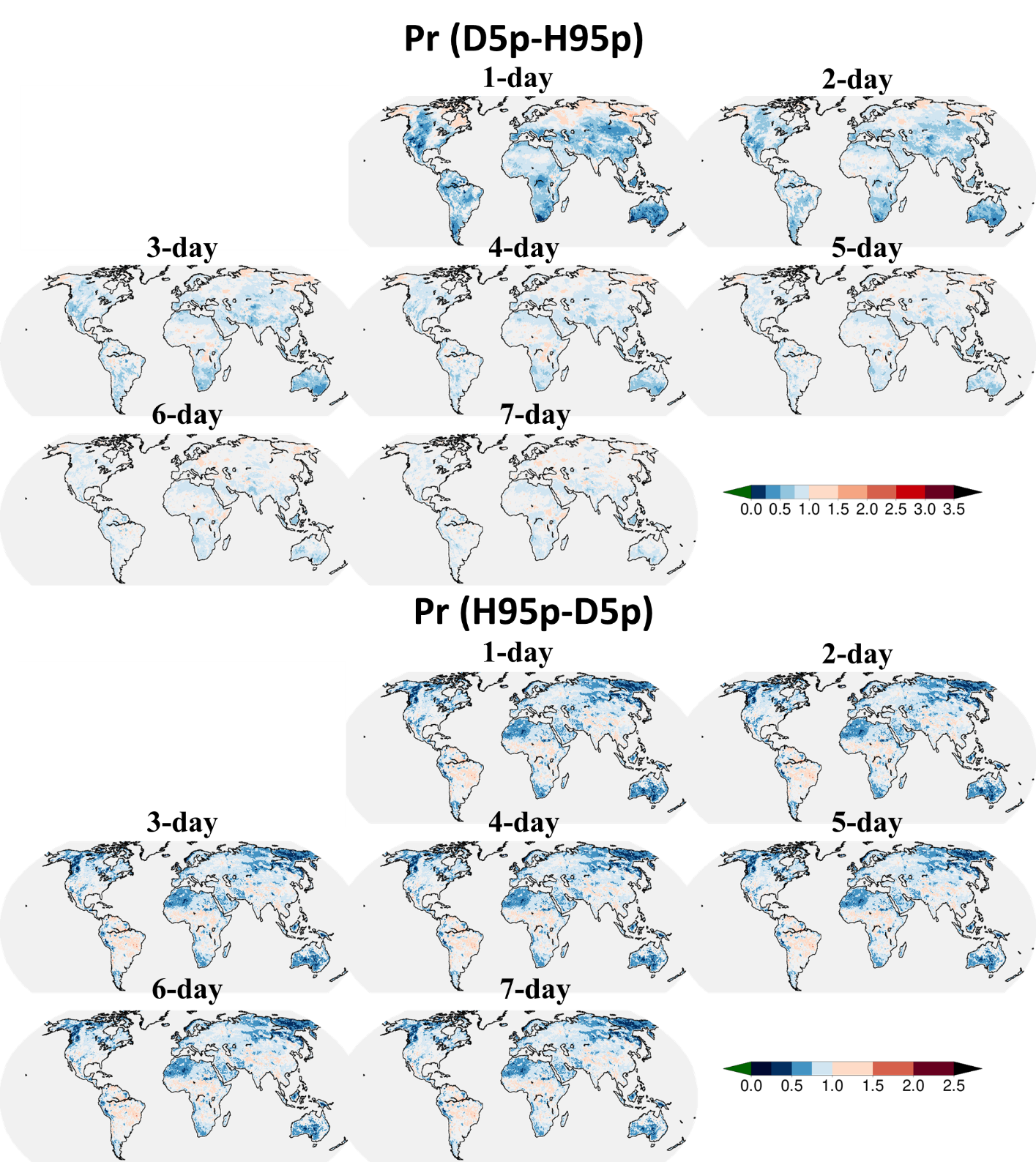
 Supplementary Figure 16** Role of confounders: **s**ame as in Figure 15 but for the dry-to-hot (D5p-H95p: top panel) and hot-to-dry (H95p-D5p: bottom panel) event cascade with 5^th^ percentile of root-zone-soil-moisture (RZSM), and 95^th^ percentile of daily maximum 2m air temperature (Tmax) as threshold.

**
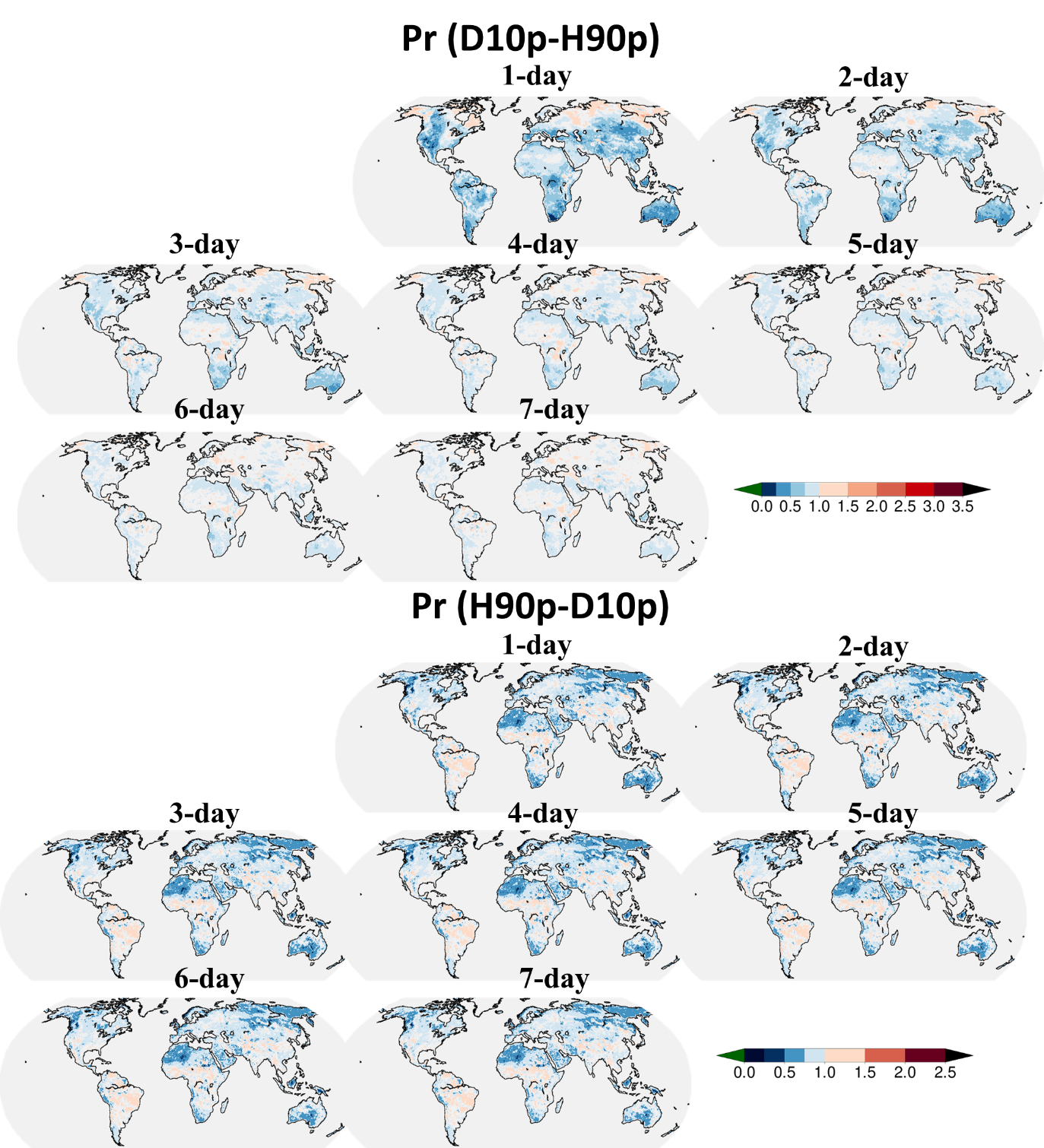
 Supplementary Figure 17** Role of confounders: same as in Figure 15 but for the dry-to-hot (D10p-H90p: top panel) and hot-to-dry (H90p-D10p: bottom panel) event cascade with 10^th^ percentile of root-zone-soil-moisture (RZSM), and 90^th^ percentile of daily maximum 2m air temperature (Tmax) as threshold.**
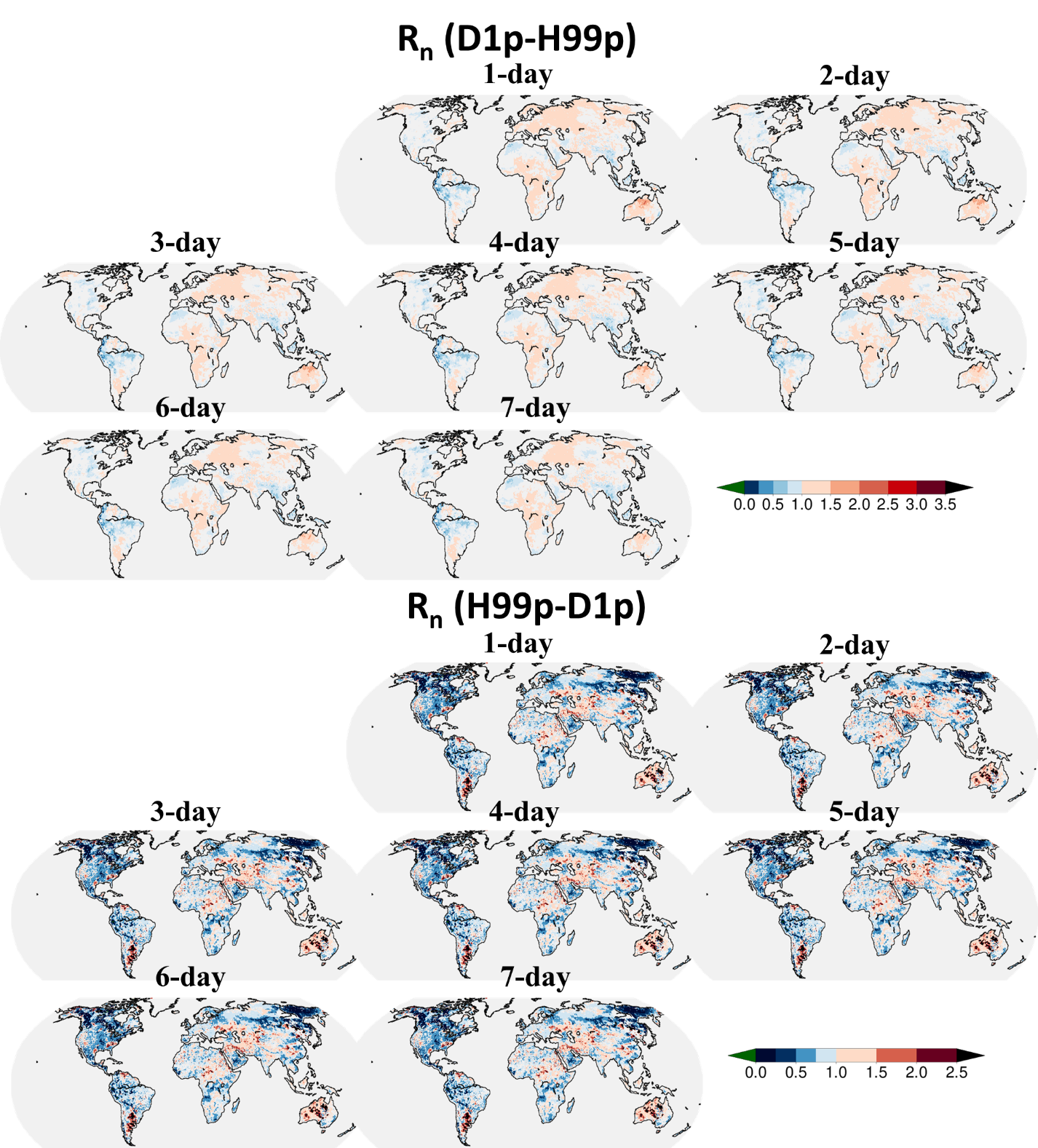
 Supplementary Figure 18** Role of confounders: spatial distribution of statistically significant (at 5% significance level) odd ratios (*exp(β)*) corresponding to net-radiation (Rn) anomalies calculated by fitting the logistic regression model for the dry-to-hot (D1p-H99p: top panel) and hot-to-dry (H99p-D1p: bottom panel) event cascade with 1 percentile of root-zone-soil-moisture (RZSM), and 99^th^ percentile of daily maximum 2m air temperature (Tmax) as threshold for a lag of 1 to 7 days.

**
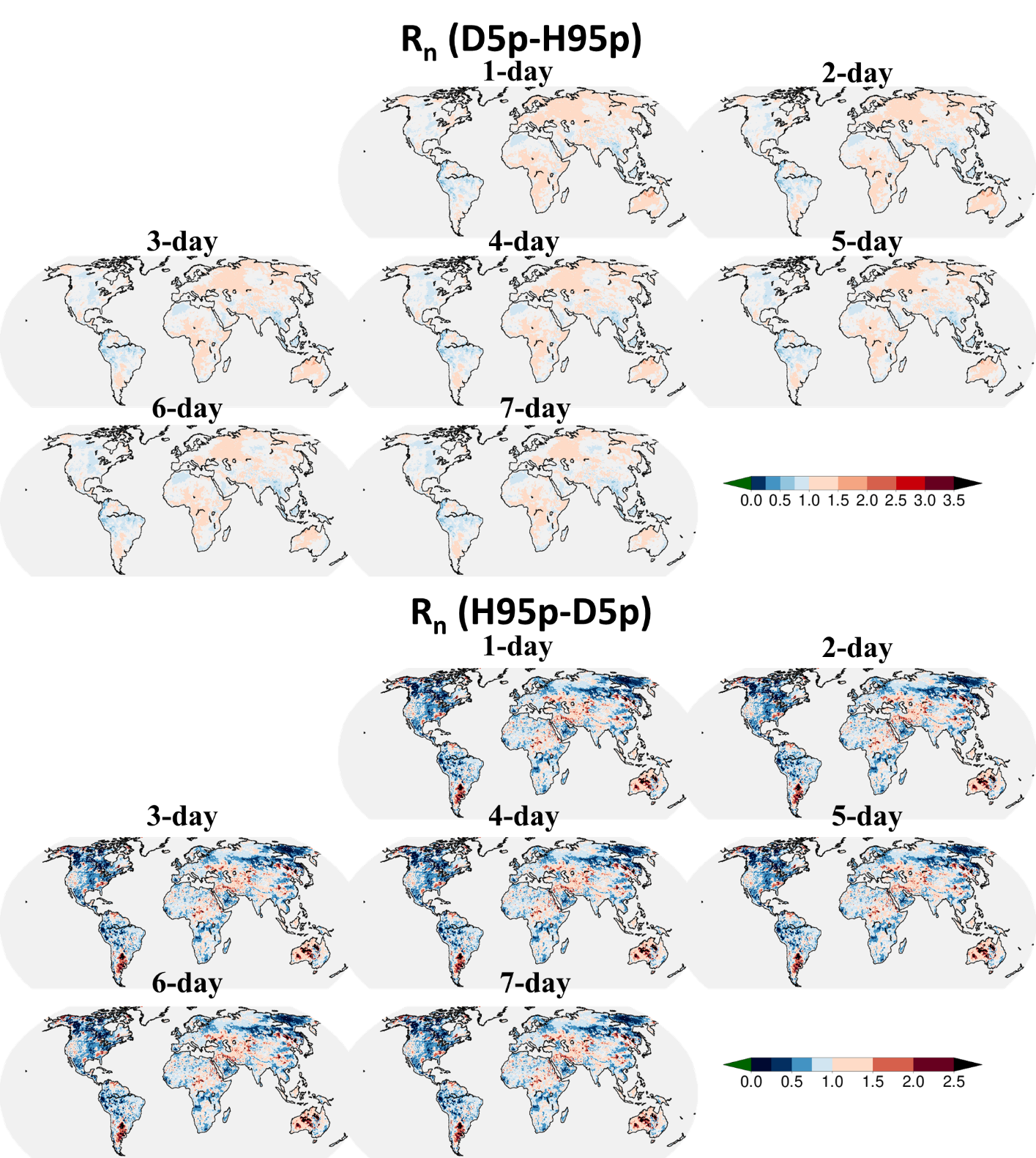
 Supplementary** **Figure 19** Role of confounders: same as in Figure 18 but for the dry-to-hot (D5p-H95p: top panel) and hot-to-dry (H95p-D5p: bottom panel) event cascade with 5^th^ percentile of root-zone-soil-moisture (RZSM), and 95^th^ percentile of daily maximum 2m air temperature (Tmax) as threshold.**
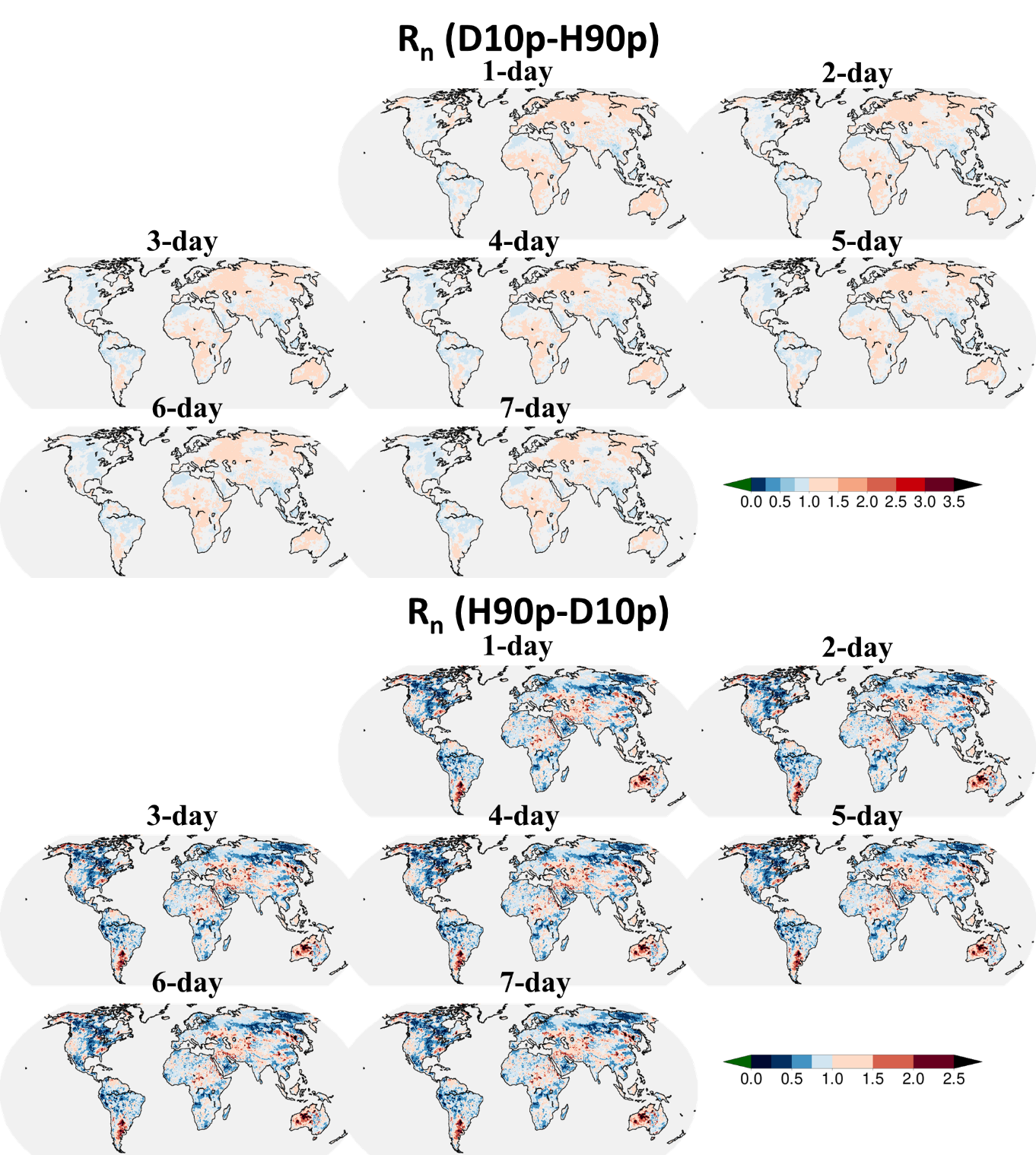
 Supplementary** **Figure 20** Role of confounders: same as in Figure 18 but for the dry-to-hot (D10p-H90p: top panel) and hot-to-dry (H90p-D10p: bottom panel) event cascade with 10^th^ percentile of root-zone-soil-moisture (RZSM), and 90^th^ percentile of daily maximum 2m air temperature (Tmax) as threshold.

**
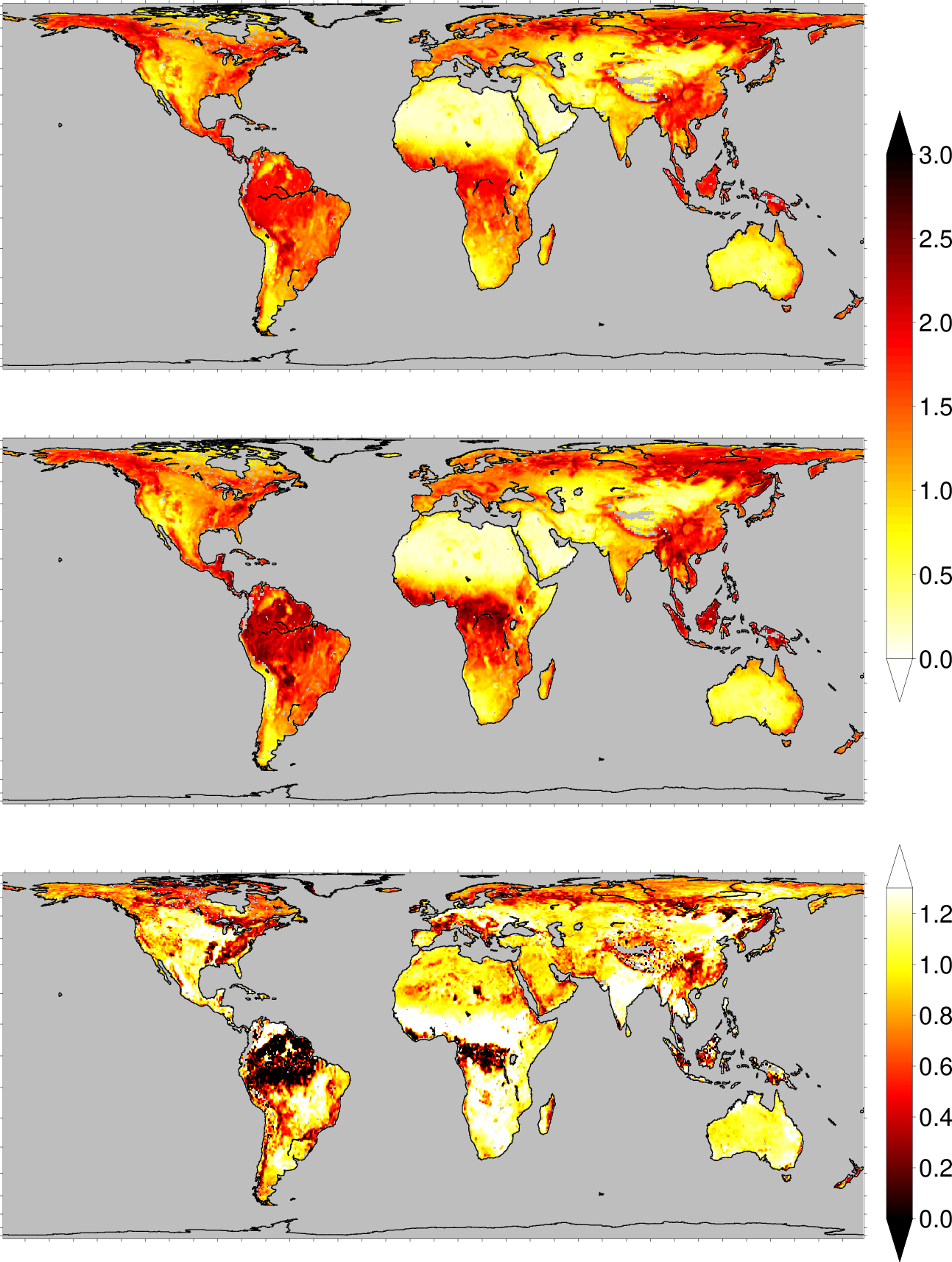
**

**Supplementary Figure 21** Estimates of vegetation optical depth (VOD)**,** Spatial distribution of mean VOD^midnight^ (top panel) and VOD^midday^ (middle panel) and isohydricity (bottom panel) calculated for the period 2003-2018.

**Supplementary References:**

1. Lu, J., Sun, G., McNulty, S. G. & Amatya, D. M. A Comparison of Six Potential Evapotranspiration Methods for Regional Use in the Southeastern United States1. *JAWRA Journal of the American Water Resources Association* **41**, 621–633 (2005).

2. Yuan, W. *et al.* Increased atmospheric vapor pressure deficit reduces global vegetation growth. *Science Advances* **5**, eaax1396 (2019).

3. Martínez-Vilalta, J., Poyatos, R., Aguadé, D., Retana, J. & Mencuccini, M. A new look at water transport regulation in plants. *New Phytologist* **204**, 105–115 (2014).

4. Roman, D. T. *et al.* The role of isohydric and anisohydric species in determining ecosystem-scale response to severe drought. *Oecologia* **179**, 641–654 (2015).

5. Konings, A. G. & Gentine, P. Global variations in ecosystem-scale isohydricity. *Global Change Biology* **23**, 891–905 (2017).

6. Richter, H. Water relations of plants in the field: some comments on the measurement of selected parameters. *Journal of Experimental Botany* **48**, 1–7 (1997).

7. Crow, W. T. *et al.* An observing system simulation experiment for Hydros radiometer-only soil moisture products. *IEEE Transactions on Geoscience and Remote Sensing* **43**, 1289–1303 (2005).
